# A Fully Automated Microfluidic Femtosecond Laser Axotomy Platform for Nerve Regeneration Studies in *C. elegans*

**DOI:** 10.1371/journal.pone.0113917

**Published:** 2014-12-03

**Authors:** Sertan Kutal Gokce, Samuel X. Guo, Navid Ghorashian, W. Neil Everett, Travis Jarrell, Aubri Kottek, Alan C. Bovik, Adela Ben-Yakar

**Affiliations:** 1 Electrical and Computer Engineering, University of Texas, Austin, Texas, 78712, United States of America; 2 Mechanical Engineering, University of Texas, Austin, Texas, 78705, United States of America; 3 Biomedical Engineering, University of Texas, Austin, Texas, 78705, United States of America; Brown University/Harvard, United States of America

## Abstract

Femtosecond laser nanosurgery has been widely accepted as an axonal injury model, enabling nerve regeneration studies in the small model organism, *Caenorhabditis elegans*. To overcome the time limitations of manual worm handling techniques, automation and new immobilization technologies must be adopted to improve throughput in these studies. While new microfluidic immobilization techniques have been developed that promise to reduce the time required for axotomies, there is a need for automated procedures to minimize the required amount of human intervention and accelerate the axotomy processes crucial for high-throughput. Here, we report a fully automated microfluidic platform for performing laser axotomies of fluorescently tagged neurons in living *Caenorhabditis elegans*. The presented automation process reduces the time required to perform axotomies within individual worms to ∼17 s/worm, at least one order of magnitude faster than manual approaches. The full automation is achieved with a unique chip design and an operation sequence that is fully computer controlled and synchronized with efficient and accurate image processing algorithms. The microfluidic device includes a T-shaped architecture and three-dimensional microfluidic interconnects to serially transport, position, and immobilize worms. The image processing algorithms can identify and precisely position axons targeted for ablation. There were no statistically significant differences observed in reconnection probabilities between axotomies carried out with the automated system and those performed manually with anesthetics. The overall success rate of automated axotomies was 67.4±3.2% of the cases (236/350) at an average processing rate of 17.0±2.4 s. This fully automated platform establishes a promising methodology for prospective genome-wide screening of nerve regeneration in *C. elegans* in a truly high-throughput manner.

## Introduction

Acute trauma to the nervous system affects a countless number of people worldwide, though the molecular mechanisms that regulate the nerve regeneration and degeneration processes remain largely unknown. A promising avenue for studying the underlying mechanisms *in vivo* is to use a small model organism such as the nematode *Caenorhabditis elegans* (*C. elegans*). Its transparent body and simple nervous system make it ideal for optical microscopy, while its high degree of genetic homology with humans and amenability to many genetic manipulations make whole-animal, genome-wide studies feasible. The study of nerve regeneration in *C. elegans* requires the use of the nano-scale precision of femtosecond laser ablation to sever single axons (laser axotomy) without inducing extensive damage in surrounding tissue. This method has been successfully adapted by the *C. elegans* community since its first demonstration in 2004 [1]–[4]. A few parallel studies have made great strides towards revealing the role of hundreds of genes and drug compounds affecting the regeneration process in *C. elegans* [5], [6], but more than 96% of the protein encoding genes still remain to be studied.

Nerve regeneration studies in living *C. elegans* are time consuming since laser axotomy requires complete immobilization of the worm so that the axon of interest can be precisely positioned within the focal volume of the laser beam. In addition, high-resolution imaging of the regenerating axons post injury is necessary, which also requires a high degree of immobilization. Among the several existing immobilization techniques, the traditional methods include the use of anesthetics [1], [2] or gluing worms to substrates [7] to ensure complete immobilization during surgery and imaging. While anesthetics might interfere with the recovery of the worms [8] and, thus, the regeneration process of the injured axons, gluing methods are also not viable options for nerve regeneration studies because the worms cannot be recovered for post-injury follow-ups. Furthermore, studies performed utilizing these methods are limited to processing a few animals per hour.

To overcome the limitations of manual techniques, new immobilization methods using microfluidic devices have been developed for both imaging [9]–[17] and surgical studies [18]–[21]. Microfluidic manipulation techniques for *in vivo* studies typically involve mechanical trapping assisted by either tapered channels [10]–[12], [22], pressurized membranes [18], [19], pressurized membranes with the addition of suction [21] or with the addition of CO_2_-induced paralysis [23], exposure to a cold (4°C) fluid to induce temporary paralysis [9], dielectrophoresis [17], or surface acoustic wave manipulation [16].

Automation of these microfluidic platforms and the related imaging and surgery processes is necessary to enable investigation of nerve regeneration in *C. elegans* at high speeds. Current approaches have their merits and potential to improve the worm processing rate. However, full automation still remains to be achieved for advancing nerve regeneration research that requires a higher level of precision and a large number of samples.

Among the first efforts towards automating laser surgery in *C. elegans*, an automated microfluidic-based system was developed for whole cell body ablation for behavioral studies [20]. This platform could immobilize nematodes with a cold fluid channel and identify each soma of interest by automatically processing a z-stack of images. The image processing algorithms performed thresholding, filtering, and automated focusing to ablate the neurons of interest. With this methodology, they could successfully ablate whole cell bodies a few microns in diameter at an average speed of 110 worms per hour or one worm every 33 seconds. This platform could potentially be very useful for behavioral assays. However, for nerve regeneration studies, this method requires further improvement in terms of precision in order to target individual 300 nm-wide axons and to distinguish the axon of interest from the other anatomical features.

In another attempt towards automated surgery, researchers have developed a semi-automated microfluidic laser surgery platform to screen chemical compounds for nerve regeneration studies [18]. In this method, the animals were loaded from multiwell plates tilted at an angle to condense the worms into the corner of each well. Once a single worm was captured from the loaded population and immobilized on the chip by the operator, the neuron of interest was then manually identified and the automation software was initiated to draw a circle with a radius equal to the preset surgery distance. In the final delicate step, the operator took over again and manually determined the intersection of the drawn circle with the axon to perform the laser axotomy process using a 0.75 NA objective. Although this semi-automated platform required a human intervention in each step of the process, it allowed the operator to perform on-chip axotomies at a speed of 20 seconds per worm. The paper did not, however, discuss whether these numbers included the failure rate of the automation and did not explore the effect of human intervention in detail.

The full automation of these complicated processes still remains unmet and requires advances not only in the image processing but also in microfluidic device design to enable high-speed and repeatable axotomies. This task becomes especially difficult when considering our goal of studying not only axonal regrowth, as carried out in most other *C. elegans* studies, but also the reconnection of the regrowing axon to the severed distal end and its fusion for functional recovery [24], [25]. Studying molecular mechanisms behind axonal reconnection in *C. elegans* is important to find treatments for restoring axonal integrity through fusion. Recent studies in mammals showed that hydrogels and certain chemical treatments could encourage axonal fusion with distal axons at the cut site if applied to the injury quickly [26], [27]. The study of axonal reconnection and fusion in *C. elegans* requires high precision laser axotomy using a high-NA, oil-immersion objective, which makes the automation process even more difficult.

Towards achieving our goals we have built and rigorously tested a fully automated microfluidic platform that can immobilize single worms from a whole population preloaded into the device and perform precise laser axotomies at a very high speed using a 1.4 NA, oil-immersion objective. We achieved this goal by completely redesigning our previous microfluidic immobilization method [19] to enable repeatable and rapid immobilization of individual worms automatically. We previously demonstrated that a microfluidic immobilization technique based on a deflectable membrane could guarantee the degree of immobilization required to perform precision laser axotomies [19]. However, this first device required manual interventions to reduce errors in immobilization orientation and the number of worms trapped at a given time that limited automated identification of the worm body and surgery on neurons of interest. To achieve full automation, we redesigned a completely new device using this immobilization concept and are able to achieve repeatable and rapid nanoaxotomy of nematodes in a fully automated manner. Utilizing novel image processing algorithms, the platform can successfully target 300 nm-wide axons with a sub-micron resolution. The automated microfluidic platform is now capable of loading a single worm from a large population, immobilizing the isolated worm, identifying the location of the worm, neuron of interest and relative location of axon, overlapping the tightly focused femtosecond laser spot on the axon of interest, and ablating it with sub-micron resolution, all in an automated manner.

## Experimental Procedures

### Device fabrication methods

**Figure 1:**
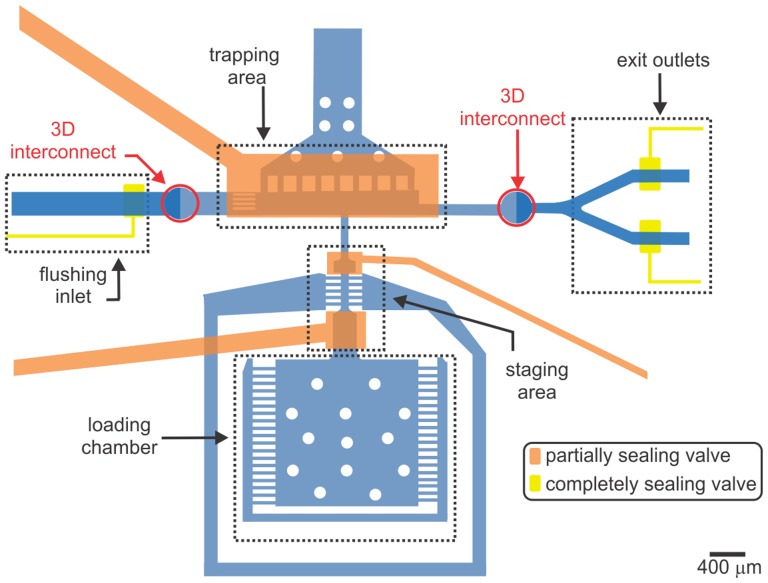
Axotomy chip overview. A schematic top-view of the microfluidic chip. The loading chamber is used to house the animals throughout the automation process. The staging area isolates a single worm from the whole population kept in the loading chamber and sends it to the trapping area for immobilization and axotomy. The T-shaped trapping area with a sieve structure enables rapid immobilization of single worms. 3D interconnects enable access to the fully sealing valves to enhance the repeatable localization of worms against the sieve structure in the trapping area by completely stopping the flow to the exit outlets. After each axotomy, the flushing inlet guides the flow to take the worm through one of the outlets.

Standard soft-lithography techniques, with some modifications, were used to fabricate the two-layer microfluidic device described here [28]. The bottom layer that transports the *C. elegans* is hereafter termed the "flow layer," and the top layer that both immobilizes the worms and actuates the valve structures on the chip when pressurized will be referred to as the “control layer”. Photoresist patterns, produced using Mylar masks (CAD/Art Services), were used to create the molds for all polydimethylsiloxane (PDMS; Sylgard 184, Dow Corning Corp) microfluidic structures. To begin, we first pattern the sieve structures by spin-coating SU-8 3005 onto a 4" silicon wafer to a thickness of 8–10 µm. The sieve structures are the arrays of short flow outlets located in the trapping, loading, and staging areas shown in Figure 1. We then coated SU-8 2025 atop the sieve structures at a thickness of 30–35 µm and created the remainder of the flow layer mold by alignment and exposure through a second photomask using a mask aligner (MA6/BA6, Suss MicroTec). The mold for the control layer was fabricated with combined patterns of a positive resist (AZ 50XT, Applied Electronic Materials) and a negative resist (SU-8 2025) [29]. Reflow of the positive resist at 125°C for 4 min was performed to create semi-circular channel cross-sections. The wafers with the developed SU-8 molds were modified with a fluorinated silane (SIT8173.0, Gelest Inc.) that served as a release agent. All resist film thicknesses were verified with a stylus profilometer (Dektak 6M, Veeco).

Fabrication of control layer PDMS involved the following steps: 1) Thoroughly mixing of the resin with the crosslinker at a ratio of 10∶1 (resin:crosslinker), 2) degassing the mixture in a vacuum chamber at room temperature, 3) pouring the degassed mixture onto the control layer mold to a thickness of ∼5 mm, 4) degassing again, and finally 5) curing at 75°C in an oven for 30 min. Creation of the flow layer involved spin-coating PDMS onto the flow layer mold at 1700 rpm for 33 s and allowing it to rest at room temperature for 5 min. This process resulted in a uniform ∼50 µm-thick PDMS flow layer and an approximately 20 µm-thick PDMS membrane covering the top of the SU-8 features. The PDMS on the flow layer mold was then partially cured on a hot-plate at 70°C for approximately 10 min. The thick control layer was aligned and bonded on top of the partially cured flow layer mold with the aid of a stereoscope. To improve the bonding strength between the two PDMS layers, the wafer was placed in an oven at 65°C overnight. After peeling the two-layer PDMS device from the mold, we punched holes through the PDMS at the inlets and outlets to make external fluidic connections. The device was then bonded to a 25×50 mm #1.5 cover slip using an oxygen plasma treatment and then placed in an oven at 65°C for 4 h to enhance the PDMS-glass bonding. Sterile polyethylene tubing (Intramedic) was connected to the device using 22 ga. stainless steel couplers (Instech Solomon) inserted into the punched PDMS holes.

### Opto-mechanical setup

The laser axotomy setup incorporates custom optics to deliver femtosecond laser pulses for surgery into a home-built epi-fluorescence microscope (Figure S1 online) [19]. We carried out axotomies using a train of femtosecond laser pulses at a center wavelength of 802 nm generated at a repetition rate of 1 kHz (Spitfire, Spectra Physics). The beam energy was measured with an energy meter (PJ10, Ophir) prior to performing all axotomies and adjusted with two sets of half wave plates/cube beam-splitters pairs. An objective lens with a high numerical aperture (NA = 1.4) tightly focused the laser beam to an estimated 1/e^2^ spot size of 620 nm [3].

Automated microscopy was performed with a 5× air objective (Plan-Apochromat, NA = 0.16, Zeiss) and a 63× oil-immersion objective (Plan-Apochromat, 63×, NA = 1.4, Zeiss). For fluorescence imaging of green fluorescence protein (GFP) labeled axons, a mercury arc lamp (XCite 120, EXFO) provided the excitation light source passing through a FITC filter set (Semrock). A three-axis translation stage made of individual actuators (LTA-HS, Newport) and operated by a single controller (ESP301-3, Newport) positioned the samples. These stages could translate at up to 5 mm/s with a minimal incremental motion of 100 nm and a lateral resolution of 35 nm (achieved after backlash compensation). High precision positioning was performed by a three-axis piezoelectric actuator (MAX 302, Thorlabs) with a minimal theoretical step size of 25 nm and a travel range of ±10 µm for each axis. A CCD camera (1392×1040 pixels with 6.45 µm pixel size, CoolSnap ES, Photometrics) captured the images with fields of view (FOV) of 1.8×1.34 mm^2^ at 5× magnification (1.29 µm/pixel, 1.88 µm resolution at 500 nm) and 143×107 µm^2^ at 63× magnification with 1.4 NA (102 nm/pixel, 214 nm resolution at 500 nm).

For controlling the device flow layer, pressurized external fluid chambers controlled by three-way solenoid valves (Lee Company, LHDA0521111H, respectively) were coupled to the chip via a manifold (LFMX0510418). These chambers contained M9 buffer solution (22 mM KH_2_PO_4_, 22 mM Na_2_HPO_4_, 85 mM NaCl, 1 mM MgSO_4_, in dH_2_O). To minimize debris, all M9 buffer was passed through 1.2 µm in-line filters (Acrodisc, Pall Corp.) prior to entering the microfluidic device. Valves were independently actuated with a multichannel amplifier (Automate Scientific) that was controlled with a DAQ card (USB6501, National Instruments).

All automation steps, including stage positioning, valve actuation, and image processing, were performed with a custom-written LabVIEW (National Instruments) program.

### *C. elegans* maintenance

*C. elegans* were maintained at 16.5°C on NGMSR (Streptomycin-Resistant Nematode Growth Medium) agar plates seeded with HB101 *E. coli* bacterial culture using standard procedures [30]. We studied the regeneration (axonal regrowth and reconnection to their distal ends) on one of the touch receptor neurons – the anterior lateral microtubule (ALM). Depending on the orientation of the immobilized worm, the axotomy was performed on either left or right ALM neuron (ALML or ALMR). We used the strain SK4005: *zdIs5* (P*mec-4::gfp*) + *lin-15*(+) I, which expresses GFP in the six touch receptor neurons [31]. Populations of age-synchronized worms were prepared by collecting and isolating embryos following hypochlorite treatment. Developmental synchronization resulted in a low degree of variability in worm's body length which significantly improved identification rates of target neurons. Gravid adults were lysed with a small volume of a 2∶1 mixture of sodium hypochlorite and 4 M sodium hydroxide, and the collected eggs were suspended in M9 buffer overnight on a rocker to aerate. The embryos hatched overnight and were arrested in the L1 stage until food was reintroduced. The L1 larvae were then placed on agar plates and allowed to grow for 48 hours, at which point the larvae had grown into worms at the young- to mid-L4 stage and could be collected for use.

### Laser axotomies and post-surgery imaging on agar pads

To compare the overall effects of on-chip axotomies with a traditional approach, we also performed manual axotomies on worms immobilized on agar pads with anesthetics. Agar pads were prepared by sandwiching 0.3 mL of melted 4% agar between two microscope slides that were then pulled apart upon cooling to create a flat and uniform surface. For anesthetization, we transferred worms with a capillary tube filled with 10 µL of 10 mM levamisole solution onto the center of the agar pad. A coverslip (18×18 mm, no. 1.5) was placed on top of the worms just prior to laser axotomy. All manual axotomies were performed on the same optomechanical setup used for automated surgery. Subsequent imaging of recovering worms was performed on agar pads using an Olympus microscope (BX-51) with a 60×, NA = 1.42, oil immersion objective. A statistical analysis of the reconnection data was conducted using the Fisher's Exact Test.

### Microfluidic chip priming and preparation for axotomies

We primed the device for experiments by first introducing M9 solution into the flow layer inlets and letting it pass through the chip and exit from the outlets for ∼5 min. This priming procedure helped to eliminate any residual bubbles within the tubing. All of the flow layer inlets were pressurized to ∼135 kPa. After priming the flow layer with M9 solution, we closed all outlets with metal plugs and pressurized all control valves to ∼205 kPa with DI water for 15 min. This procedure replaced the air within the valves with DI water by forcing the air to diffuse out into the PDMS.

Before performing each set of axotomies, we experimentally determined the axial (*z*) offset between the focal plane of the camera and the beam waist location of the laser. To determine this offset, we first brought the closest surface of the glass slide into camera focus and created a small laser ablation spot on the surface by delivering a train of 300 pulses at 10 nJ/pulse. This ablation spot on the surface of the slide appears as a small dark circle surrounded by a lighter circle. The objective was then moved in 0.25 µm steps towards the ablation spot until the smallest two-dimensional (2D) ablation profile was observed. The resulting location refers to the smallest laser beam waist location; which was typically about 0.5 µm for our microscope setup. Moving deeper towards the glass slide, the Gouy phase shift of the laser beam was observed. The phase shift location refers to the axial position where the 2D ablation profile transitions from a bright to a dark color; which was typically about 0.9 µm for this setup. To achieve repeatable and physically consistent ablation on the axon, we performed ablations by defocusing 0.5–0.9 µm below the focal plane of the visible light.

## Results and Discussion

Our automated microfluidic platform is designed to perform high-resolution laser axotomies in a fast and serial manner on single worms without affecting worm viability or the need for user intervention. To simplify hardware manipulation and workflow in our optical setup and to enhance axotomy precision, we maintained a single location for optical interrogation and processed the animals serially. Unlike most other nerve regeneration studies, our system uses a high-NA oil immersion objective and well-tuned laser parameters to increase axotomy precision. As previously shown, the regeneration capability of injured axons directly depends on the precision of laser injury and extent of damage [3]. When the damage to the surrounding tissues is minimized, the severed axons could not only regrow, but also reconnect to their distal partners via fusion [24], [25] to recover full functionality [1] thus restoring neural circuitry. Further, high precision requires minimizing the aberrations by directly immobilizing the worms on the cover glass without an intervening layer of PMDS, air, or liquid. Other nerve regeneration studies, that have relied on more relaxed focusing parameters using low-NA air objectives, mainly focus on axonal regrowth [18], [32]. Our system enables a more complete understanding of neuroregenerative processes by enabling to study the reconnection processes in addition to regrowth.

To enable high-precision laser axotomies, we have previously established a new methodology of pressurizing and deforming a membrane to immobilize worms directly against the optical interface during surgery [19]. By eliminating an extra layer of PDMS or dead channel volume between worms and the cover glass, our design offered ideal optical focusing and accuracy of ablation for studying the complete regeneration process.

In our current design, adopting the same immobilization methodology we redesigned the chip into a unique configuration to enable its full automation. The new chip includes five distinct parts (Figure 1): (1) a loading chamber for housing a population of up to 250 worms, (2) a staging area for isolating a single worm from the population in the loading chamber and delivering it to the trapping area, (3) a trapping area designed to ensure repeatable and rapid immobilization of single worms near to the focused laser spot, (4) 3D interconnects for enabling transition to completely sealing valves, and (5) flush exit outlets for transitioning processed worms to an off-chip location.

In the following sections, we detail our design considerations and how each design specification contributed to fulfilling these considerations. We then explain the automation sequence that includes both automated actuation of valves and on-chip flow, as well as image analysis of captured worms and their neurons. The description of the custom-developed software and corresponding hardware list is given in File S1. This supplementary document also includes the instructions for the software download and installation.

### Microfluidic device design to enable automation

A key design consideration when developing an automated approach to perform laser axotomies in *C. elegans* is to optimize the delivery of a single worm from a large, on-chip population into the trapping area while minimizing the degree of spatial variability in the trapped position of the worm. More specifically, the ability to repeatedly trap only a single worm at the same location is essential to improving the rate at which the worm can be successfully targeted by the image processing software.

To reduce ambiguity in axonal reconnection data, other design considerations included: overcoming delivery challenges such as trapping multiple worms, trapping a worm in a folded configuration (see Figure S2 online), and sending a non-axotomized worm into the pool of axotomized worms. With these challenges in mind, we designed a new chip with three new components: 1) a staging area to isolate individual worms from preloaded population, 2) a T-shaped configured trapping area for fast and repeatable worm trapping, and 3) 3D interconnects to incorporate fully sealing valves.

**Figure 2:**
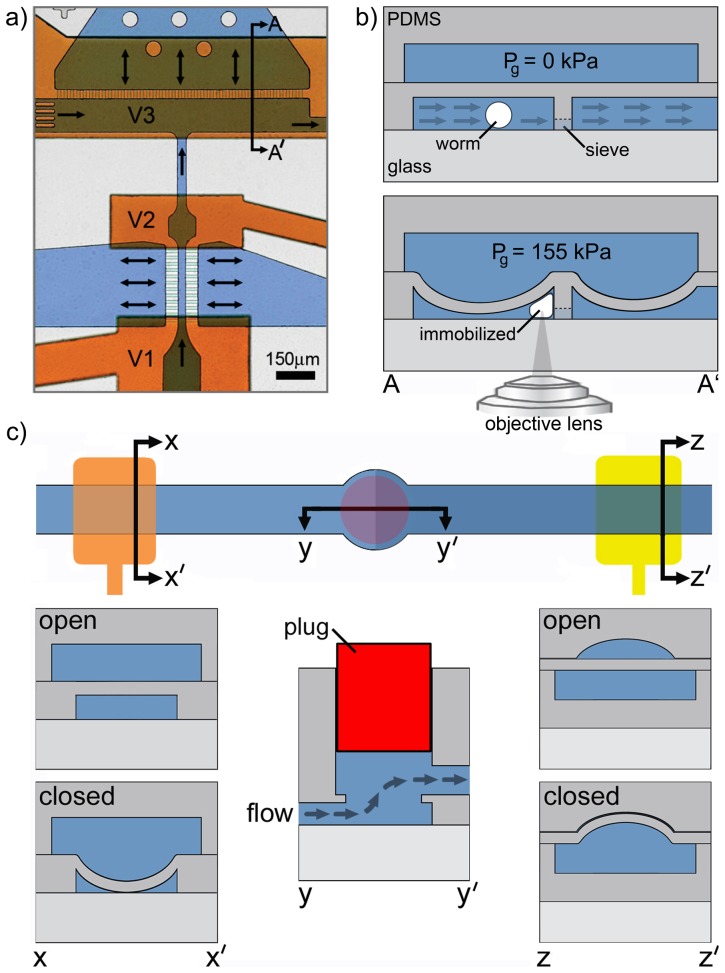
Flow direction in T-shaped staging/trapping area and 3D interconnects. (a) Optical image of a dye-filled microfluidic device with black arrows indicating the direction of fluid flow. Orange dye fills the control layer and the blue dye identifies the flow channels. The loading chamber holds pre-loaded worms before their serial transportation into the staging/loading area. The synchronized actuation of valves V1 and V2 in the staging/loading area allows loading of a single worm against the microfluidic sieve in the immobilization zone. A partially sealing trapping membrane, actuated by valve V3 in the control layer, then physically immobilizes the worm for laser axotomy. (b) Schematic cross-section referring to the sectioning arrows A-A' in (a) that shows the flow direction through the sieves during membrane deflection, location of the worm in the trapping area during delivery and after membrane deflection, and the relative heights of the microfluidic sieve and channel within the immobilization zone. The objective is placed underneath the glass interface. Avoiding any additional PDMS layers in the optical pathway eliminates optical distortion for imaging and ablation. (c) Top view and cross-sectional view (y-y') of a 3D interconnect that enables transitioning the flow from the 1^st^ layer (controlled by partially sealing valves) into the 2^nd^ layer controlled by completely sealing valves. A metallic pin (red) is plugged half-way into the through hole that connects the layers. The cross-sectional view of a partially sealing valve is given along x-x' while a completely sealing valve is shown along z-z'.

1. *Staging area*: In the co-linear configuration of our previous design [19] we observed that worms, arriving at the trapping area at very high speeds, would occasionally result in multiple worms being trapped or some worms being lost when transporting away an axotomized worm. To isolate a single worm from a pre-loaded population of worms and avoid trapping of multiple worms, we positioned a staging area before the trapping area. The new staging area incorporates two partially sealing gate valves (V1, V2) separated by a narrow channel that allows for the containment of only one L4 life-stage worm and stops other worms from entering (Figure 2a). The main staging channel is 30 µm×35 µm with a length of 600 µm, the approximate dimensions of an L4-stage worm [33]. We have previously shown that a deflected PDMS membrane could almost completely seal a 30 µm-deep, 120 µm-wide rectangular channel, leaving small gaps in the bottom corners of the channel [19]. Accordingly, the staging channel beneath the gate valves (V1, V2) was designed to be 120 µm wide to prohibit staged or non-staged worms from passing through the actuated valves.
2. *T-shaped configured trapping area*: The new T-shape trapping area design enables the automated delivery and trapping of single worms in the desired location (Figure 2a). Automatic identification of the neuron of interest in the trapped worm requires processing of high-resolution images within a small FOV. It is therefore important to immobilize the worm in a location close to the desired FOV for finding the neuron rapidly using a single step image acquisition. In our previous co-linear trap design, positioning the worm at the desired location repeatedly was difficult because the worm could easily move past this part of the device and we had to reverse the flow to reposition the worm [19]. In an effort to circumvent this problem, we and other groups incorporated complex manipulation methods for fine-positioning the worms using, for example, side channels [18], [19]. However, such complex manipulations required many manual device interventions. The proposed T-shaped channel configuration of the trapping area eliminates the need for such complex manipulations by providing two major advantages. First, the structure provides a repeatable immobilization location by injecting the worm straight close to the middle of the sieve structure. Second, the channel shape allows for perpendicular decoupling of the injection and flushing channels, which permits a reliable and straightforward worm ejection after axotomy. Worm's entire body is pushed against an array of short and narrow flow outlets that form the microfluidic sieve structure (Figure 2b). The pressure drop across these channels immediately straightens the delivered worm into an elongated body position just before actuation of the trapping membrane.
3. *3D interconnects*: Microfluidic valves actuated in channels with rectangular cross-sections do not blockage the channel flow completely, even at very high valve pressures. While the staging area configuration took advantage of the leaky nature of these valves, such structures downstream from the trapping area resulted in inconsistent positioning of worms during immobilization. We found that worms delivered to the center of the trapping area were better immobilized than off-centered worms. Bending membrane of the trapping area could restrict better the worm's whole-body when positioned at the center of the trapping area. This step was critical for minimizing worm motility and its motion due to pharyngeal pumping and led to an improvement in ablation accuracy. This type of positioning error could be overcome with valves that completely sealed the exit flow channels. Valves actuated in channels with hemispherical cross-sections have been known to seal completely. However their fabrication would necessitate a thin layer of PDMS [9], [20] or a control layer between the worm and the cover glass in the trapping area [34]. The presence of this additional PDMS layer in the optical path would introduce an index of refraction mismatch and spherical aberrations at focusing distances beyond 30–40 µm, reducing the system's effective NA and compromising the precision of both imaging and surgery. To avoid the additional PDMS layer, we diverted the flow to another microfluidic flow layer and connected both flow layers using new 3D interconnects. Interconnects were created on both sides of the trapping area by punching thru-holes at overlapping microfluidic structures within the first and second layers, as show in Figure 2c. To prevent media from flowing out of the device from the punched thru-hole, a permanent metallic plug (Figure 2c) was inserted into the thru-hole at a depth that it did not interfere with flow within the interconnect. Fabricating the fully sealing valves in a different layer than the axotomy layer and connecting them with a permanent 3D interconnect, eliminated optical distortion and thus degradation of the imaging and ablation quality of the system.

These three features simplified the image processing-based approach for identifying anatomical features of the worm and sped up the process of automated analysis.

Our final design consideration related to the capture of un-wanted debris during device operation. Despite efforts to remove particulate matter from the worm suspension in M9 buffer prior loading worms into the device, we still found an accumulation of a small amount of microscopic particles within the chip during the automated process. To prevent the sieve structures from clogging in the staging and trapping areas, which negatively impacted chip performance and could eventually lead to total chip failure, we built an array of staggered filter structures with gaps ranging from 10 µm down to 5 µm at the entrance of each flow channel [35]. Additionally, we fabricated an array of pillars spaced 30 µm apart that allowed for the passage of worms into the loading chamber, but blocked a large fraction of debris from entering the trapping area and clogging the sieve structures in the staging and trapping areas.

### Progression of valve actuation, flow, and worm manipulation processing

The automation software, run by LabVIEW, controls the external solenoid valves maintaining flow into the device and the on-chip valves to synchronize the automation steps. Specifically, it stages worms for serial processing, traps them individually for laser surgery, and controls the remainder of flow throughout the device (Movie S1). While the worms are trapped, the program synchronously works with image processing algorithms to perform laser axotomies on trapped worms, as described in the next section (Movie S2). For the entire duration of automated operation, a constant head pressure of ∼15 kPa is used to continually drive flow through the loading chamber and move worms into the staging area.

**Figure 3:**
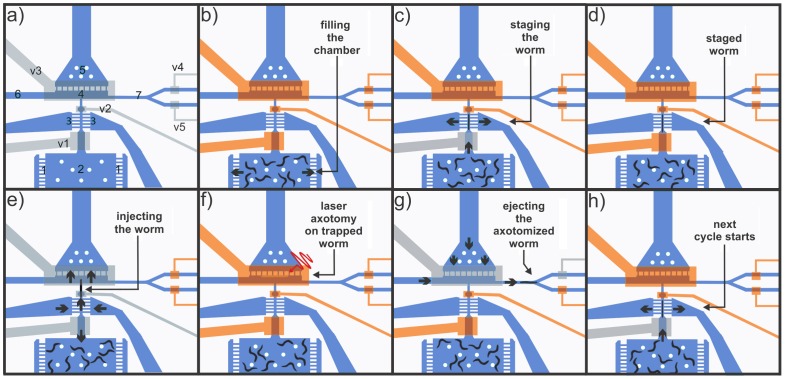
Automated progression of valve actuation and flow. (a) A schematic representation of the chip, showing the valves, channels, and chambers/areas indicated by their reference numbers. Valves V1 and V2 are used to stage and inject individual worms serially into the trapping area *(4)*. Valve V3 serves as the immobilizing membrane. Valves V4 and V5 are used to open and close the ejection and flush channels, respectively. The ejection channel is used to deliver worms that have been successfully axotomized to a recovery area, and the flush channel is used to jettison worms that failed at any step of the automation process. Two-way flow through the side channels *(3)* in the staging area aid in serial staging and injection of worms into the trapping area. A two-way channel *(5)* in the trapping area *(4)* allows the flow through the microfluidic sieve structure to push the worm against the sieve structure during loading and away from the sieve structure during unloading of the worm along with the one-way side channel *(6)* leading to the trapping area. (b) Filling the loading chamber *(2)* with a population of worms by opening side channels *(1)* and closing all other valves and channels. (c) Staging of a single worm by opening valve V1, closing valve V2, and pushing the worm into the staging channel where flow is directed out through the side channels *(3)*. (d) Preventing additional worms from entering the staging area by closing valve V1. (e) Injecting the worm into the trapping area by opening valves V2 and V3 and reversing the flow in the staging side channels *(3)*. Valve V1 is also opened to eliminate other worms entering the staging area prematurely. (f) Immobilizing the worm by closing valve V3 in a cyclical pumping manner (Movie S1) and then performing automated axotomy on the trapped worm using image processing algorithms. (g) Transporting the successfully axotomized worm into the recovery area through the ejection channel *(7)*. (h) Starting the next cycle by loading the next worm in the staging area.

The sequence of valve and flow progression at each step during automation is shown in Figure 3. The numbering of valves and chambers/channels is provided in Figure 3a. We first load a population of worms into the device by blocking all flow channels except the small flow exits *(1)* in the side of the loading chamber *(2)* (Figure 3b). After the loading chamber has been filled with worms, the gate in the staging area is used to withdraw a single worm and inject it into the trapping area by opening and closing the two valves located at the front (V1) and back (V2) of the staging zone (see Figs. 3c–3d). An array of small channels *(3)* on both sides of the staging channel acts as a sieve-like fluidic path to direct the worm between valves V1 and V2 during staging.

Once a worm is located in the staging area, it is injected into the trapping area *(4)* by reversing the flow through the staging sieve with a head pressure of ∼65 kPa (back into the staging channel and opening valves V1, V2, and V3 for 1000–1200 ms (Figure 3e). This valve sequence pushes the worm against the sieve *(5)* in the trapping area, preparing it for trapping and surgery. Reversing flow in the staging sieve and opening valve V1 during this injection period also helps to prevent worms within the loading chamber from prematurely entering the staging area. During the trapping phase, valve V3 is actuated in a cyclical manner (close, open, and close) to avoid unfavorable worm folding (Figure S2 online) as the membrane began to immobilize the worm. We found that this trapping approach could improve worm viability and the success rate of the automation.

Immediately after immobilizing the loaded worm and recognizing its body centroid via image processing algorithms, the system moves the translation stage automatically to align the center of the worm body within the center of the FOV. After switching the objective from 5× to 63×, the software proceeds to position the worm for the axotomy, and axon ablation is executed (Figure 3f). Details of the image processing algorithm and axon ablation are described in the following section.

Immediately after the axotomy, the system switches back to the 5× objective and the software simultaneously opens valves V3 and either V4 (flush) or V5 (ejection) (Figure 3g). The image algorithm software makes an informed decision as to whether or not the outcome of each individual automation step during trapping and axotomy is successful or not. If the outcome is negative, the corresponding worm is flushed through the waste channel immediately after the unsuccessful step. Successfully axotomized worms are collected through the ejection channel. An external solenoid valve is simultaneously actuated to reverse flow through the sieve structure in the trapping area and deliver fluid through the one-way-flow flush channel, both with a head pressure of ∼135 kPa. After the valve sequence rapidly removes the worm from the trapping area and delivers it through either the flush or the ejection channel, the next cycle starts with staging the next worm (Figure 3h, just as in Figure 3c). This automated process is repeated until axotomies are performed on the desired number of worms from the loaded sample population, and any remaining worms ate then removed from the chip by opening all on-chip valves and reversing the flow through the sieve structure in the trapping area. The entire process of staging, trapping, and axotomy requires, on average, about 17 s/worm.

### Image processing methodology for automated identification of neurons and targeting for laser axotomy

We developed a custom image processing methodology that performed axotomies automatically on GFP-labeled mechanosensory ALM neurons (Movie S2). A pair of these neurons runs anteriorly along both sides of the worm from somas located close to the worm's centroid. The automation software uses a four-step image processing procedure to automatically identify the neuron, locate the target on the desired axon, and perform laser axotomy for injury. The software is also able to detect and flush away worms that are not immobilized in a way that allows for the image processing algorithms to pursue a successful axotomy. A flow chart describing the whole automation process, including image processing steps, is given in Figure S3 online. It is important to note that Step 1 in the flow chart involves all the valve actuation steps to trap a single worm, including staging, injection and trapping, in addition to the image processing step that are discussed below.

#### Step 1: Identification of the worm location and its centroid (Figure 4)

**Figure 4:**
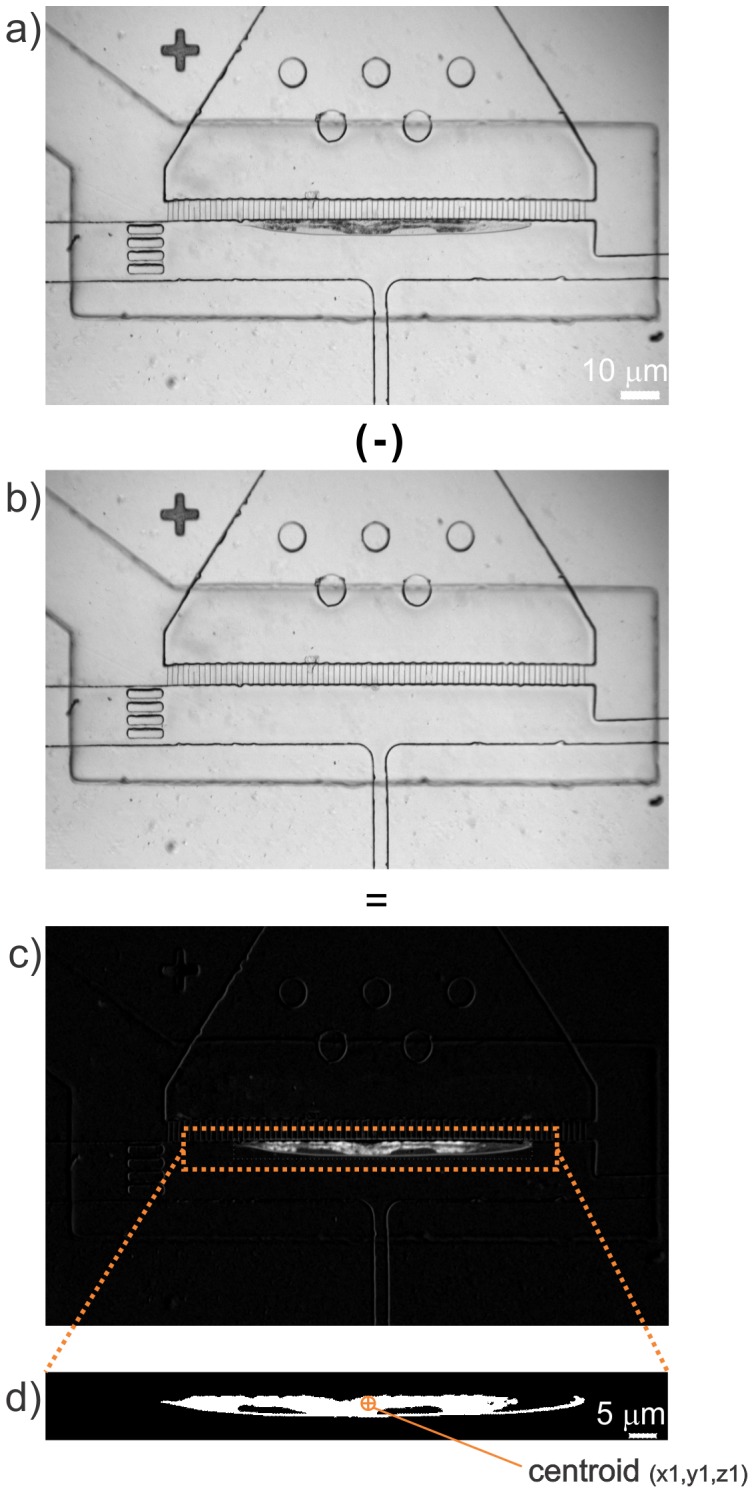
Image processing methodology for identifying the worm location within the trapping area (Step 1). (a) Bright-field image of an immobilized worm. (b) Background image of the trapping area with the same FOV as shown in (a) with the trapping membrane fully actuated. The image in (b) is then subtracted from the image in (a), resulting in the image given in (c). (d) Image thresholding and noise filtering is then used to generate a high degree of contrast so that the centroid of the worm can be accurately located.

Once a worm has been immobilized, which can occur anywhere within the trapping area (Figure 4a), it is necessary to accurately identify the centroid of the worm. The knowledge of the centroid location helps to verify that the worm is immobilized in a correct position and also provides valuable information on the location of the neuron of interest, the ALM in this study. The stereotyped neuroanatomy of *C. elegans* enables to accurately position the high-magnification, small FOV in the vicinity of the desired neuron for the subsequent automation steps, where fine focusing of the target axon and ablation occurs. By processing the low-magnification (5×), bright-field images of the trapping area, the software finds the relative location of the worm within the trapping area. The image processing involves background subtraction and thresholding. Before trapping a worm, the software saves a baseline image of the same FOV without a worm but with the trap membrane deflected (Figure 4b). By subtracting the baseline image from the snapshot of the trapped worm, the background is removed and only the worm remains in the resultant image (Figure 4c). The program next extracts a pre-defined region of interest (ROI) from the resultant image, based on the perimeter of the trapping area and then an image-thresholding filter is applied to enhance contrast (Figure 4d). To eliminate noise in the processed image, a particle filter is used to remove all features spanning areas smaller than 300 pixels. If the detected center of the worm is found to be near the edge of the ROI, the trapped worm is flushed (rejected) and the software proceeds with staging the next worm. This simple subtraction algorithm eliminates the need for complex and time-consuming pattern recognition algorithms, and functioned well for any worm size.

#### Step 2: Detection of a neuronal cell body (soma) in the small FOV (Figure 5)

**Figure 5:**
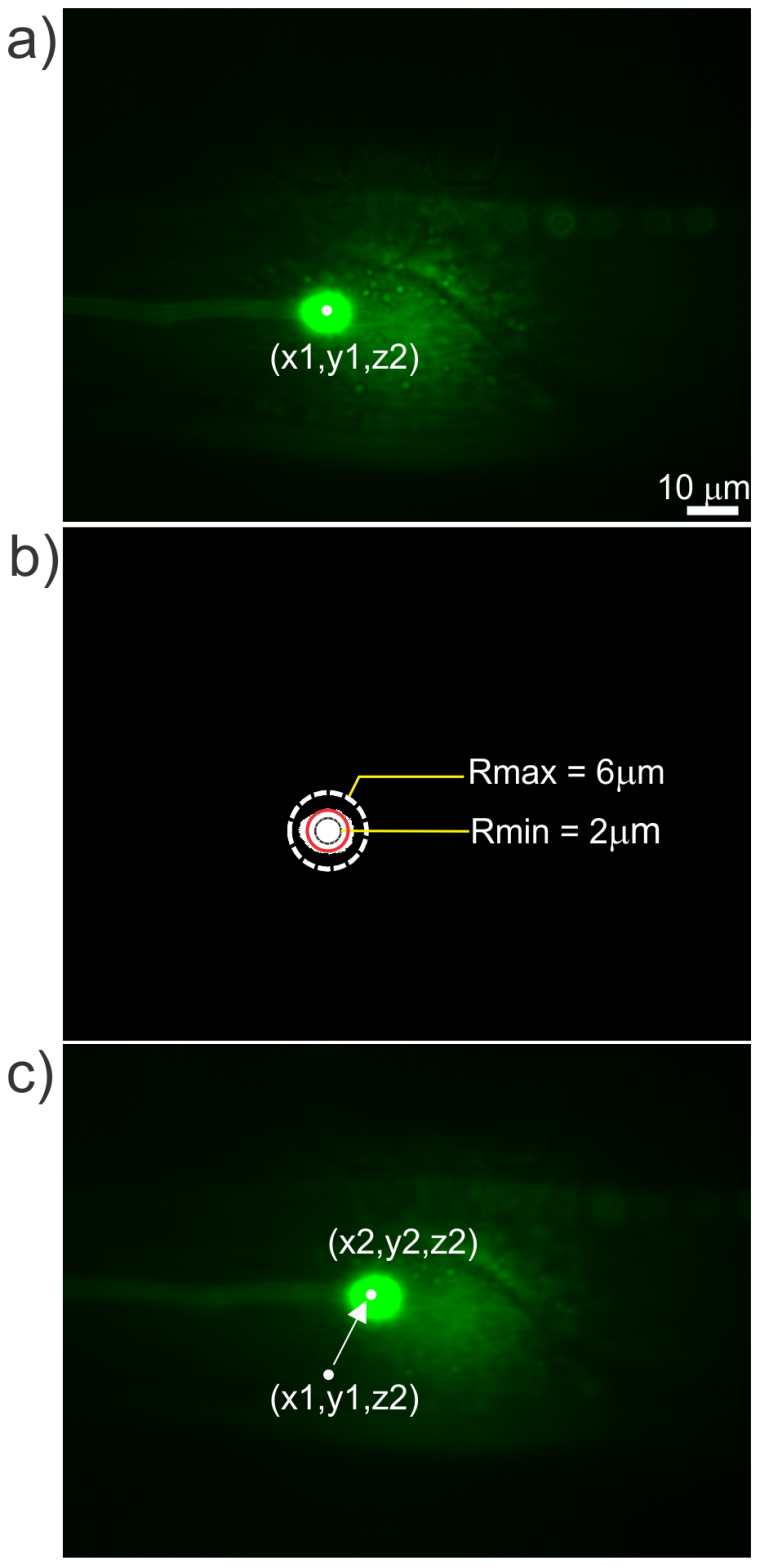
The circular object detection methodology to detect a cell body in the field of view (Step 2). (a) Fluorescence image of the detected cell body in the 63× FOV. (b) Thresholded image of the fluorescent cell body in the 63× FOV. In the thresholded fluorescence images, the automation program looks for objects with circular shapes having diameters between 2 µm (red circle) to 6 µm (dashed circle). The white object shows the detected cell body that is a little larger than the lower limit of desired size of the cell body. (c) The fluorescence image of the cell body is relocated to the location of the laser spot, which is close to the center of the FOV to aid in further image processing.

**Figure 6:**
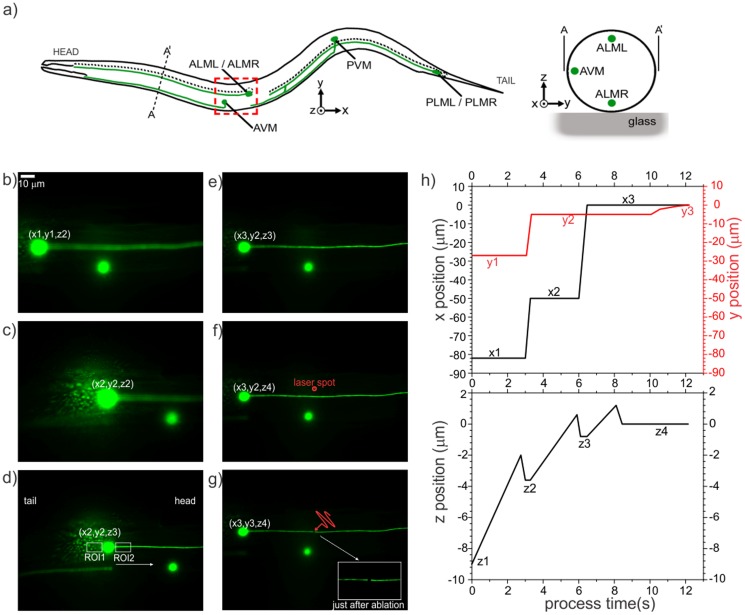
Image processing steps to verify the neuron of interest (Step 3), locate the target on the desired axon, and perform laser axotomy (Step 4). (a) A schematic of *C. elegans*, showing the relative anatomical locations of the neuron of interest (*i.e.*, ALML and ALMR). (b) Automation software finds the centroid of the worm and moves the focus proximal to the glass/worm interface at position (x1, y1, z1), as described in Step 1, and then locates a cell body using coarse focusing with the motorized stage to (x1, y1, z2). (c) After finding cell bodies in the FOV, the program brings a cell body in the vicinity of the laser spot at (x2, y2, z2). (d) The resultant z-location after performing fine-focusing on the detected cell body. To determine the relative location of the axon and distinguish the ALM from the AVM, as described in Step 3, the programs looks for straight edges in two small ROIs on the sides of the cell body. (e,f) After determining the orientation of the worm, the translation stage moves along the axon to (x3,y2,z3) and performs a quick focusing procedure on the axon at (x3,y2,z4) to obtain the best focus. (g) The piezo stage finally translates the axon to the laser spot location along the y-axis to position (x3,y3,z4) and performs laser axotomy. The insert shows a magnified view of the cut axon just after the ablation. (h) Graphs of the lateral (top) and axial (bottom) positions of the neuron of interest during the overall image analysis part of the automated axotomy process in the small FOV.

After the centroid of the trapped worm is defined and translated into the center of the FOV, the 63× objective lens moves into place and the illumination is switched to fluorescence mode. Here, finding the centroid of the worm in Step 1 ensures that there are only ALM and possibly AVM neurons present in the small FOV. Other anatomical features may have to be utilized to perform axotomies on other neurons such as the PLM. To find a cell body in the small FOV, the coarse z-stage then begins to advance the focal plane in steps of 2.5 µm into the body of the worm (Figure 5a), collecting fluorescent images at each increment and looking for circular features. Each step moves the focal plane towards either the left or right ALM neuron, reaching whichever neuron is positioned closest to the cover glass/worm interface, until a circular shape corresponding to the soma is detected. It is important to note that when we perform surgeries on ALMs, no distinction is made between the left and right ALM (Figure 6a). To find a cell body, images collected at each z-step are thresholded to a pre-determined intensity cutoff (determined empirically to be 8× the mean intensity) until a circular feature drops to a radius between 2 and 6 µm (Figure 5b). If the program cannot detect a circular feature in more than 10 iterations, it flushes the trapped worm and proceeds to stage the next worm. This cell body-locating process provides a fast method to find the neuron soma and adequately bring it to the focal plane, avoiding the need for slower fine-focusing approaches with higher resolution capabilities that are unnecessary at this stage.

#### Step 3: Verification of neuron of interest (Figs. 6d and Figure S4)

The automation software continues with verification of the neuron of interest and the axon's orientation relative to the anterior/posterior body axis of the worm. By fine-focusing on the cell body and looking for axonal extensions, the program determines if the detected cell body is the neuron of interest and finds its orientation. For fine-focusing, a z-stack of images (0.7 µm steps) is collected via piezoelectric actuator translation (Figure S4). The image with the highest variance of pixel intensity correlates to the most in-focus z-position [36], [37]. The variance of intensity in each frame 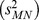 is calculated by:



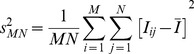
(1),where 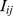 is the intensity of a single pixel in the image and 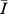
is the average pixel intensity of an image with an *M*×*N* array of pixels. Before the variance of intensity of each frame is calculated, a 2D Laplacian of Gaussian (LoG) bandpass filter [38] is convolved with each image to simultaneously reduce high-frequency noise and enhance the intensity of the axon. The LoG-filtered image is given as:



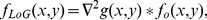
(2)where



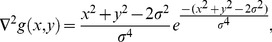
(3)


and 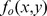 is the original unfiltered image, and σ is assigned a value of 2. After the best focus is achieved, the automation program creates two small rectangular ROIs (150×200 pixels) on the left- and right-hand sides of the cell body to determine if an axon is in the form of a straight feature within each ROI (Figure 6d). The existence of such a feature distinguishes the ALM neuron from the AVM, which is the only other possible neuron expressing GFP within the small FOV based on the known neuroanatomy and the GFP promoter used in our strain. The detection of an axon also determines the orientation of the worm in terms of its head and tail (Figure 6a); ALM neurons extend their processes towards the head in most cases. In some cases, however, they also have a processes extending towards the tail. In these cases, the brightest axon usually indicates the main axon extending towards the head. If the program cannot identify straight edges in either side of the neuronal soma, it decides that the cell body is not the neuron of interest and flushes the trapped worm and proceeds to staging a new worm.

#### Step 4: Laser axotomy (Figs. 6e–6g)

After verifying whether the cell body belongs to the neuron of interest and determining the axon location, the translation stage moves 50 µm from the soma towards the head of the nematode (Figure 6e). Then the automation program performs another fine focusing step to locate the axon within the focal volume of the laser beam along the z-direction (Figure 6f). In a manner different from the previous fine focusing step, a direct comparison of mean pixel intensities of the regions in the vicinity of axon branch is used to find the best z-location. In the final step before axon ablation, the piezoelectric actuator moves precisely in the y-direction to align the laser spot with the axon. Given that the diameter of the axons of interest is ∼300 nm, their precise ablation with a laser beam focused to a 1/*e* ^2^ diameter ablation spot of ∼620 nm requires high-precision alignment. With the 63× objective, these dimensions correspond to three and seven pixels respectively, giving a positioning tolerance for axotomy of only one pixel on either side of the axon to ensure a proper cut mainly with the center of the beam. Due to the positioning hysteresis of the piezoelectric actuators, we incorporated a closed-loop proportional control algorithm based on imaging feedback to drive the actuators to find the sub-pixel center of the axon in the y-axis. The distance (in pixels) between the axon's center and the ablation spot serves as the iterative error in the control algorithm, thereby determining the distance that the piezoelectric actuator needs to be translated. The process is repeated until the axon is positioned within ∼1 pixel from the ablation target (Figure 6g). If the program cannot achieve this goal in 10 iterations, it flushes the worm and proceeds with staging the next worm. Spatiotemporal plots of the target location on the neuron of interest relative to the focal point of the laser beam during the small FOV (63×) automated processing step, are given in Figure 6h.

### Characterization of the automated platform

#### Effect of chip manipulation on worm survivability

To determine the effect of on-chip immobilization on worm survivability, we processed a population of 20 worms without performing axotomies, using a full cycle of valve actuation (as described in Figure 3), and immobilizing each worm for 30 s with a trap pressure of 155 kPa. Control and trial groups underwent the same synchronization and cleaning procedures. The trapped worms were collected on NGMSR plates and compared with the control group. We checked the viability of the worms in each population every 24 hours and transferred worms to new NGMSR plates whenever necessary. We used the Log-Rank test to determine the difference between the viability of each group. The average lifespan of the immobilized population was found to be 17.6±4.2 days, whereas worms in the control group lived for an average of 19.3±3.3 days. No statistically significant (*p = *0.14) difference in outcome was identified for the two groups (see Figure S5 online).

#### Timing and axotomy success rates

**Figure 7:**
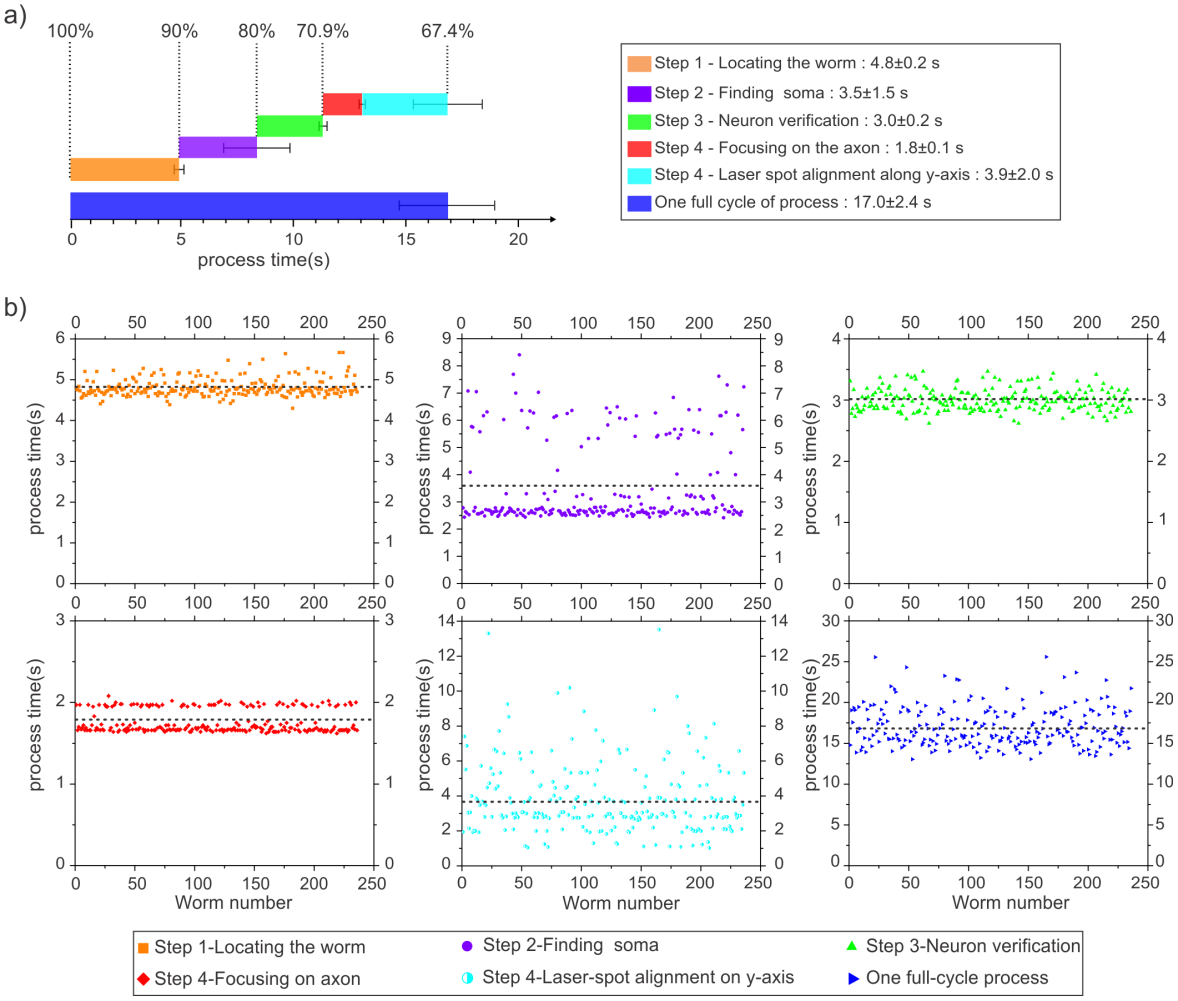
Success rate and timing performance of the automation. a) The average times required to perform a successful automated axotomy in single neuron per animal for each individual step of the automation process are indicated with colored bars. The accompanying error bars indicate the standard deviations measured from two different sets of experiments with a total number of successfully axotomized worms of n = 236. The average percentages of worms remaining (from all the processed worms, n = 350) after each step is given above each corresponding time bar at the top of the figure. b) Timing of automation steps for each successful trapping experiment (n = 236). Timing of each step for the same worm is addressed with the same worm number for each graph.

The axotomy success rate was found to be 67.6±3.2% on four separate sets of experiments, namely 236 successful axotomies among 350 processed worms (Figure S6). The standard deviation represents the error of the mean of four different sets. The time required for each individual step in the process was recorded by our automation software and presented in Figure 7. The average timing values for each step are given in Figure 7a, with error bars representing the standard deviation of timing distribution for each successful automation step. The timings of each step are presented for each individual experiment in Figure 7b. One of the main reasons for the variability in each step's timing were due to variation in the relative location of the neuron of interest to the cover glass (in the z-direction) and variation of the orientation of each trapped worm (in the x- and y-directions). The percentages along the top of Figure 7a indicate the remaining population after each step of automation.

We found Step 1 to be the second longest step (4.8 s) of the full cycle after Step 4. This step includes staging, injecting, and immobilizing the worm, along with carrying out image processing algorithms to calculate the relative location of the worm. Approximately 10% of the population was flushed through the waste outlet due to either the appearance of bubbles in the trapping area or the simultaneous processing of multiple worms.

Coarse focusing (Step 2) step took approximately 3.5 s with a rejection percentage of 10%. The main reason for rejection was the difficulty in detecting a cell body in the coarse focusing procedure. As the translation stage moved towards the cell body to identify a circular shaped object, because of the coarse steps, the stage would sometimes pass beyond the cell body in between the steps and miss locating a cell body in the FOV. This step also showed a high variation in timing because of the variation of the variation of the cell body location with respect to initial positioning of the stage (Figure 7b). This step was very effective in coarsely locating the cell body quickly after switching from low NA to high NA imaging.

Step 3 resulted in flushing a moderate number of worms (9.1%) when the edge detection could not verify the neuron of interest due to a weak axonal signal in most of these cases.

The longest step was Step 4 (5.7±2.1 s) to enable precise laser axotomy that required performing time-consuming fine-focusing process on the axon and high-resolution alignment of the laser spot with the axon along the y-axis. While Step 4 was the longest one, it was the step with the highest success rate. Only a small number of worms (3.5%) did not go through a successful ablation in this last step. If the system could not move the axon sufficiently close to the focal plane in the z-axis based on the pre-defined number of iterations and stage adjustment ranges, the system would be unable to find the axon target along the y-axis due to the out-of-focus background signal. The high variation of timing for the laser spot alignment could be mainly related to the inherent hysteresis of the open-loop piezo actuator while positioning the laser spot with respect to the axon. This issue can easily be overcome by replacing the actuator with a strain-gauge based controllers. The second minor issue was related to the variation in the background signals and/or morphological differences between worms.

On average, the total automation time to process each animal was found to be 17.0±2.4 s/worm among the n = 236 successful axotomies obtained in four separate runs (Figure S6). We could use each microfluidic chip for approximately 20 hours before sieve structures in the trapping and staging area became overly clogged with debris. The debris could come from tubings, metal couplers, small worms getting stuck in the sieve structures, and PDMS particulates. We cleaned the devices after 3 hours of operation with a 5% of bleach solution in deionized water. At some point, approximately after 20 hours of total operation time, flow in these regions of the device became compromised to the point of rendering the device unreliable.

#### Axonal reconnection success rates after automated on-chip surgery

**Figure 8:**
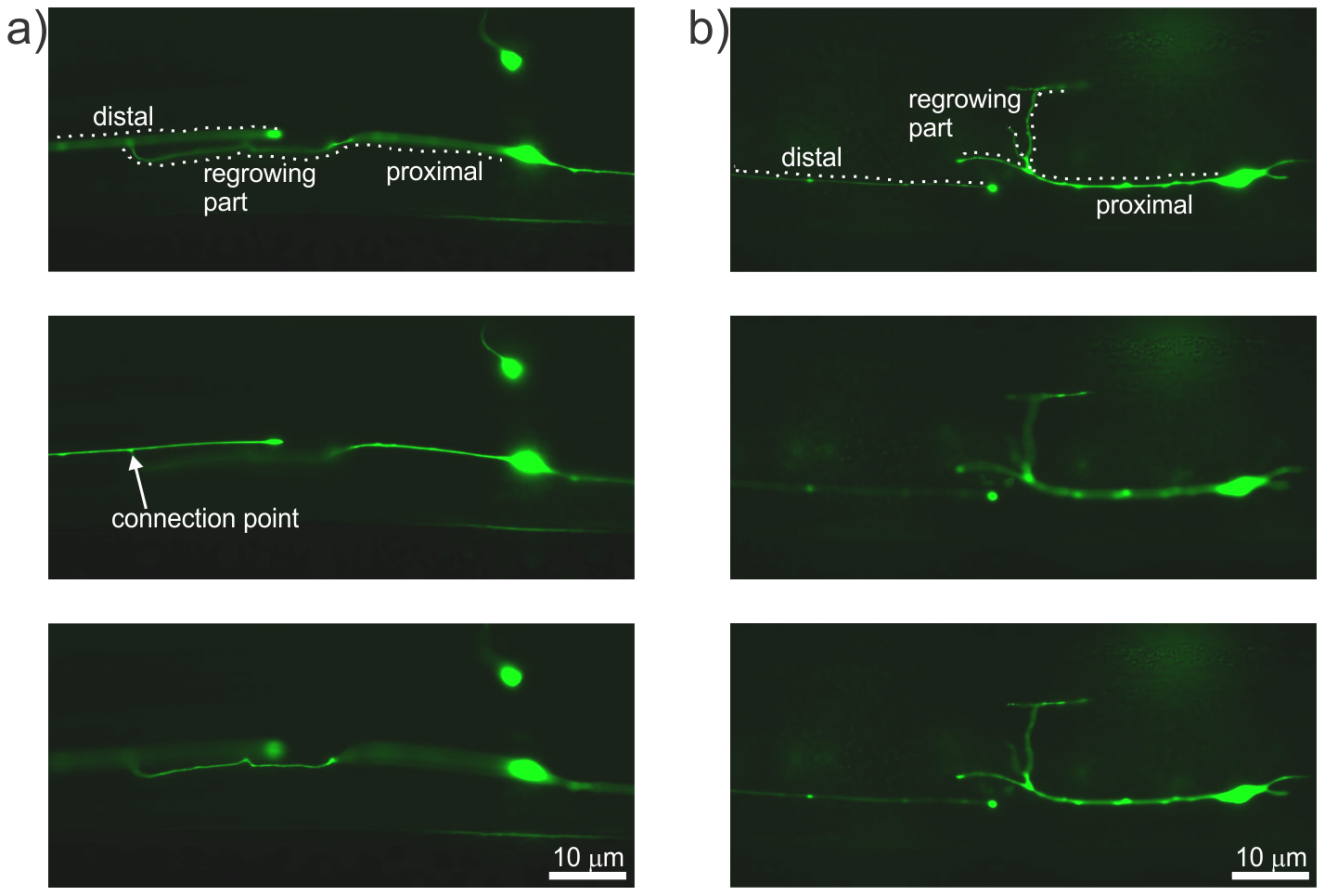
Imaging of regrowing axons of interest 24 hours after surgery. a) Images at three different z-locations showing an example of reconnection with the connection point of distal and proximal ends indicated by the arrow. b) Images at three different focal planes showing regrowth with a lack of reconnection, as evidenced by the severed ends taking paths in different focal planes. The fragmentation and beading in the distal part shows the lack of reconnection.

**Figure 9:**
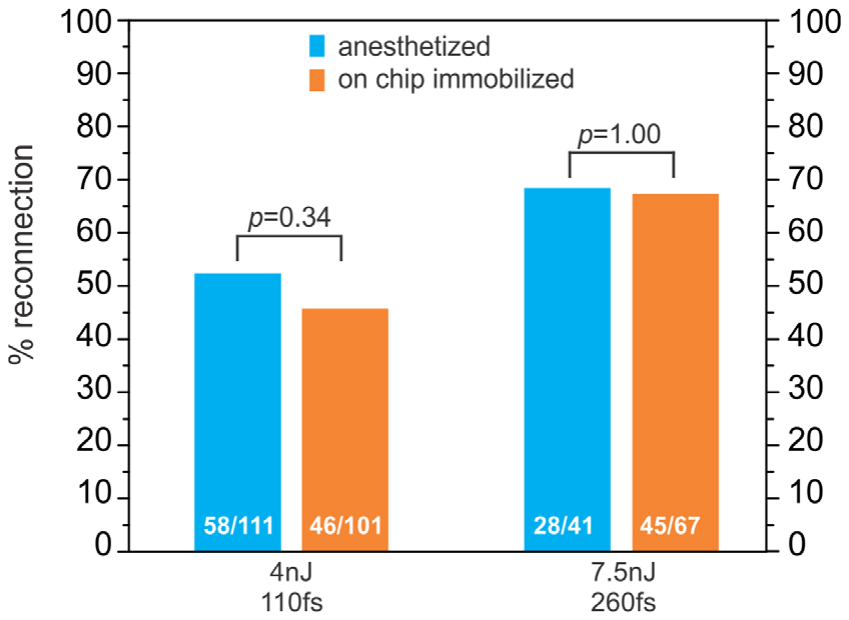
Axonal reconnection results. Axonal reconnection rates of the ALM neuron for two different laser conditions (300 pulses of 4 nJ pulse energy, 110 fs pulse-width and 300 pulses of 7.5 nJ pulse energy, 260 fs pulse width). The results show no statistically significant differences between automated axotomies on the chip and axotomies performed on agar pads using anesthetics. The numbers of reconnected neurons are given inside each bar. Statistical significance is determined using Fisher's exact test; p≥0.05 in both cases.

To determine axonal reconnection rates following on-chip laser axotomies, we collected the axotomized worms on freshly seeded agar plates and imaged the axotomy site for signs of regrowth and reconnection after 24 h of post-surgical recovery at 16.5°C (Figure 8). We specifically looked for successful reconnections in the ALM neurons after automated on-chip axotomies and compared them to the results obtained by manual ablation on agar pads. Two primary criteria were used to describe robust reconnection: (1) proximal re-growth trajectories intersecting the distal axon and (2) a lack of beading or fragmentation in the distal axon that normally marks the beginning of Wallerian degeneration [24]. For example, Figure 8a shows a fluorescence image of an injured axon reconnecting successfully to its distal end. Whereas regrowth with a lack of reconnection is evident by a dimmed GFP signal and by beading in the distal part of the axon (Figure 8b). We found no statistically significant differences for reconnection probabilities at two different laser ablation conditions between manually performed axotomies and those carried out with our automated approach (Figure 9). For the first ablation condition, we used a train of 300 laser pulses with a pulse energy of 4 nJ and pulse-width of 110 fs, whereas for the second condition we used 300 pulses each having an energy of 7.5 nJ and pulse-width of 260 fs. For the first ablation condition, the reconnection rate under anesthetized conditions was 58/111 = 52%, whereas the on-chip reconnection rate was 46/101 = 46% (*p* = 0.34, Fisher's Exact Test). For the second ablation condition, the reconnection rate under anesthetized conditions was 28/41 = 68%, whereas the on-chip axotomy reconnection rate was 45/67 = 67% (*p* = 1.00, Fisher's Exact Test).

## Conclusions

We have successfully built, demonstrated, and validated a fully automated microfluidic platform for performing laser axotomies in living *C. elegans*. The autonomous system successfully cut axons in average 67% of the worms loaded into the microfluidic device, and the process of automated targeting and axotomy required 17 seconds for each worm. We found no statistically significant differences for reconnection probabilities between axotomies performed manually using anesthetics and our automated approach for two different ablation conditions.

We successfully combined efficient and accurate image analysis techniques with this microfluidic platform to perform multiple surgeries in a serial manner, with synchronized valve and flow progression facilitating rapid transport and immobilization of individual worms. The automated platform used image processing algorithms to locate and target axons for ablation at a record rate of ∼17 seconds per worm. Such rates are likely required for productive, high-throughput screening studies, thereby providing an opportunity to perform genetic screening in a reasonable timeframe to identify the molecular mechanisms involved in inhibiting or promoting nerve regeneration and degeneration.

The automated platform provides approximately one order of magnitude improvement over manually performed axotomies when considering study of a single population. Including slide preparation, surgery, and unloading of the animals, a single set of manual axotomies on 15 worms, in our lab, takes at least 1.5 hours; which gives an average of 3 hours per population of 30 worms. In a conservative calculation, our automated system can currently process 30 worms (a single population) in a single run of 16 minutes. This time includes the loading of the population (2–3 minutes), successful axotomies on 30 worms (8–10 minutes), and cleaning/priming of the microfluidic chip after processing the worms (2–3 minutes). Comparing this average speed (16 minutes per population) to the speed of manual axotomies (3 hours per population), the automation currently provides at least 11 times improvement over the manual approaches.

For future work, this axotomy chip could be connected downstream of the multiwell population delivery device we recently developed to significantly improve its throughput capacity [29]. In a parallel effort, we are also developing a high speed, automated confocal imaging system that can perform fast three-dimensional imaging of the regrowing axons. With the combined population delivery – axotomy system and the high-speed, automated confocal imaging platform, it might eventually be possible to perform a genome-wide screening for individual genetic mediators of axon regeneration within a time frame of a few years. Using the current system, we can perform axotomies on four populations of 30 worms within one day.

Finally, the presented image processing methodology can be adapted for ablation on PLM and AVM neurons with the incorporation of small changes to the algorithm in Step 3 of the automation process. Additionally, the microfluidic platform can be used as a phenotype screening platform with modifications to the image processing algorithm.

## Supporting Information

Figure S1 **Schematic of the femtosecond laser axotomy setup.** The GFP-labeled neurons of the worms are excited by a mercury arc lamp (blue lines) and imaged by a high-NA objective lens onto a CCD camera (green lines). The surgery pulses are delivered to the sample through the same objective lens after being attenuated (red lines). Legend: SH – shutter, ATR – attenuator, FL – femtosecond laser, ML – mercury lamp, MR – mirror, DM – dichroic mirror, WL – white light source.
(TIF)

Figure S2 **Folded worm in the trapping area.** The undesired orientation of the worm inside the trapping area which was overcome by actuating the trapping membrane, valve V3, in a cyclical manner during the initial trapping procedure while being pushed by the flow from the staging area.
(TIF)

Figure S3 **Automation Flowchart.** Flow chart illustrating the steps of the whole automation process, including the image processing algorithms used to find and ablate the axon of interest.
(TIF)

Figure S4 **Fine focusing methodology to verify the neuron of interest and determine the orientation of the axon.** Stack of images on the left are obtained during fine focusing using the piezoelectric actuator with a step size of 0.7 µm. The desired focal plane is determined by finding the image with the highest variance in pixel intensity. Two selected images from this stack are shown separately, one in focus (top right) and one out of focus (bottom right) to show different degrees of focus. The neuron with an axon sprouting from its side can be identified as the ALMR and the neuron without any axon can be identified as the AVM following the neuronal anatomy of *C. elegans*.
(TIF)

Figure S5 **Lifespan analysis.** The viability of worms immobilized automatically on the chip with an applied trapping pressure of 155 kPa for 30 seconds (blue) as compared to the control group (orange). (Log-Rank test, *p* = 0.14).
(TIF)

Figure S6 **Statistical analysis of the automation steps.** Four separate sets of automation experiments were pursued. Axons were severed successfully in 67.6±3.2% of the cases (n = 350). Actual number of processed worms and successfully severed worms are given inside each bar. Average full cycle process time for each set of experiments is given below the each bar in parenthesis.
(TIF)

File S1 **This supplementary document describes the automation software and the necessary hardware used for performing automated laser axotomy experiments described in the manuscript and provides the installation instructions of the software.**
(PDF)

Movie S1 **A real-time video of the automated staging, trapping, and collection of the worms in a serial manner.** Each worm isolated from a pre-loaded population and staged individually. The isolated worm is injected to the trapping area for immobilization and collected after immobilization.
(MP4)

Movie S2 **A real-time video of the fully automated axotomy.** The video shows the sequence of automated steps for cell-body finding, neuron verification, fine focusing on axon, and laser spot alignment.
(MP4)
