# Supplementary figures and images for: A Fully Automated Microfluidic Femtosecond Laser Axotomy Platform for Nerve Regeneration Studies in C. elegans
Source: PLoS One. 2014 Dec 3;9(12):e113917. doi: 10.1371/journal.pone.0113917 (PMC4254741; doi:10.1371/journal.pone.0113917)

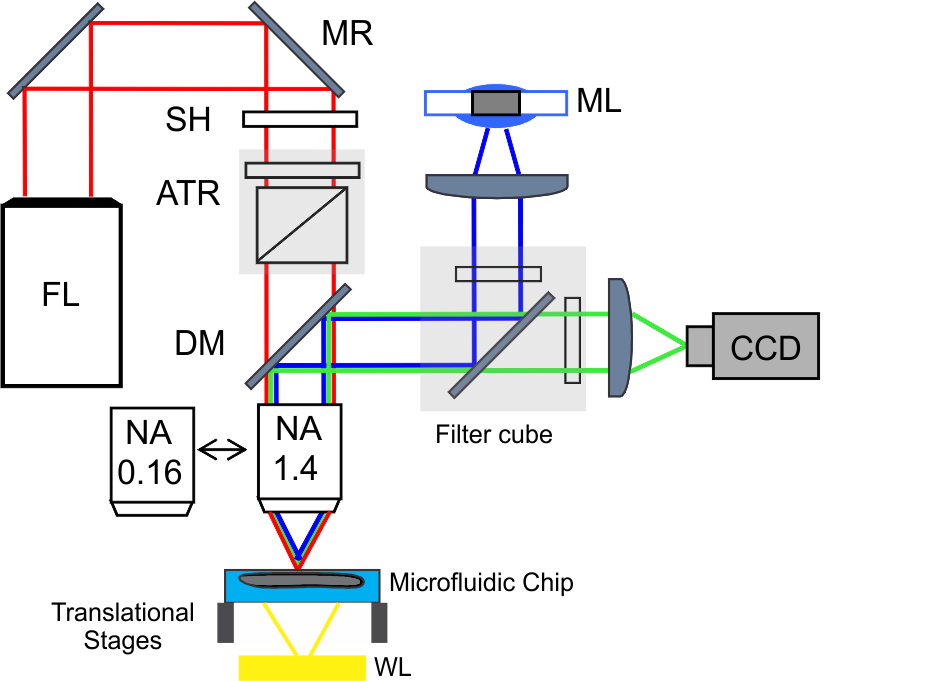

Supplement: Figure S1 — Schematic of the femtosecond laser axotomy setup. The GFP-labeled neurons of the worms are excited by a mercury arc lamp (blue lines) and imaged by a high-NA objective lens onto a CCD camera (green lines). The surgery pulses are delivered to the sample through the same objective lens after being attenuated (red lines). Legend: SH – shutter, ATR – attenuator, FL – femtosecond laser, ML – mercury lamp, MR – mirror, DM – dichroic mirror, WL – white light source. (TIF) [file pone.0113917.s001.tif]

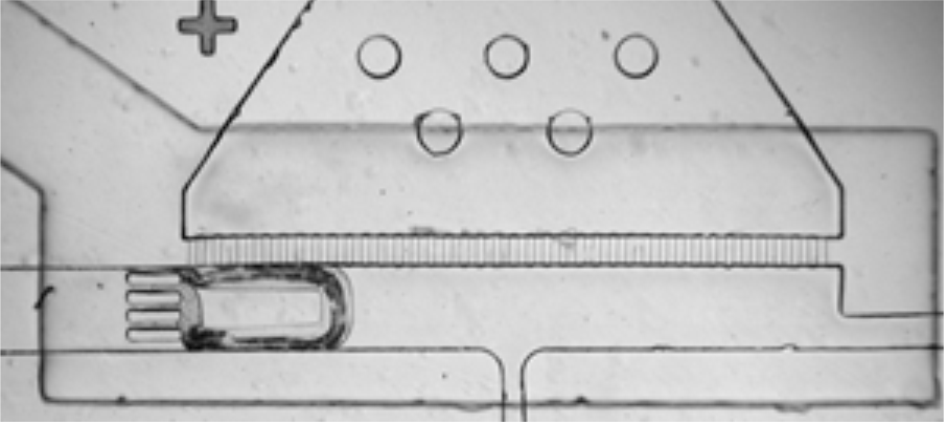

Supplement: Figure S2 — Folded worm in the trapping area. The undesired orientation of the worm inside the trapping area which was overcome by actuating the trapping membrane, valve V3, in a cyclical manner during the initial trapping procedure while being pushed by the flow from the staging area. (TIF) [file pone.0113917.s002.tif]

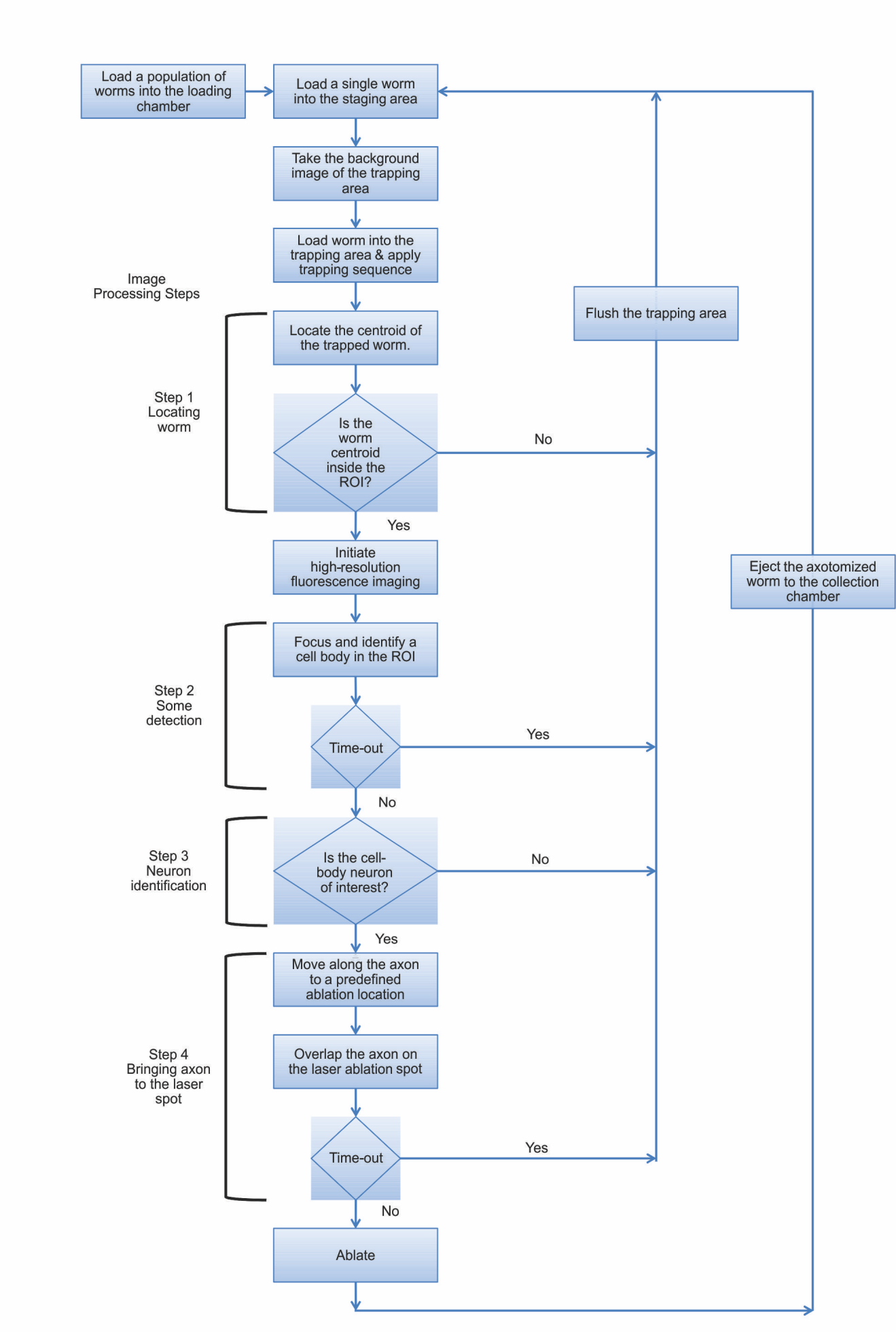

Supplement: Figure S3 — Automation Flowchart. Flow chart illustrating the steps of the whole automation process, including the image processing algorithms used to find and ablate the axon of interest. (TIF) [file pone.0113917.s003.tif]

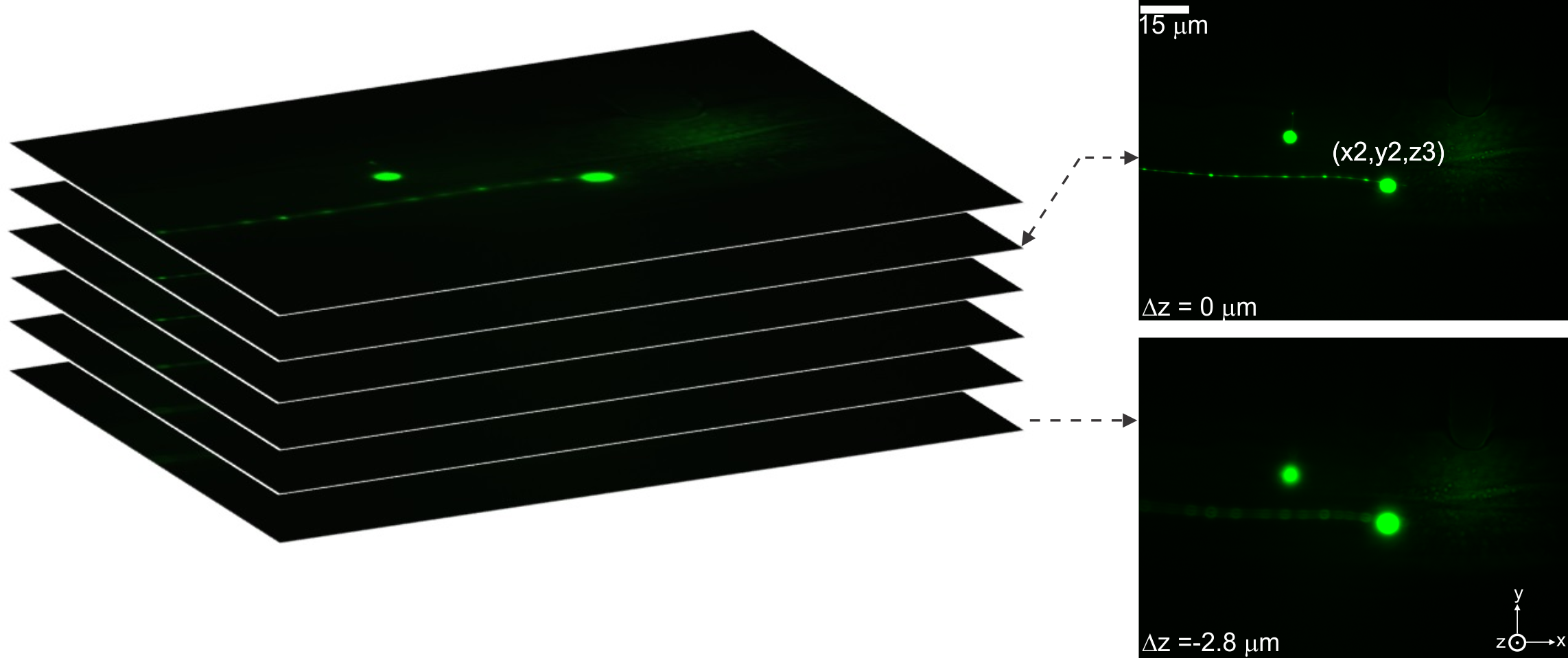

Supplement: Figure S4 — Fine focusing methodology to verify the neuron of interest and determine the orientation of the axon. Stack of images on the left are obtained during fine focusing using the piezoelectric actuator with a step size of 0.7 µm. The desired focal plane is determined by finding the image with the highest variance in pixel intensity. Two selected images from this stack are shown separately, one in focus (top right) and one out of focus (bottom right) to show different degrees of focus. The neuron with an axon sprouting from its side can be identified as the ALMR and the neuron without any axon can be identified as the AVM following the neuronal anatomy of C. elegans. (TIF) [file pone.0113917.s004.tif]

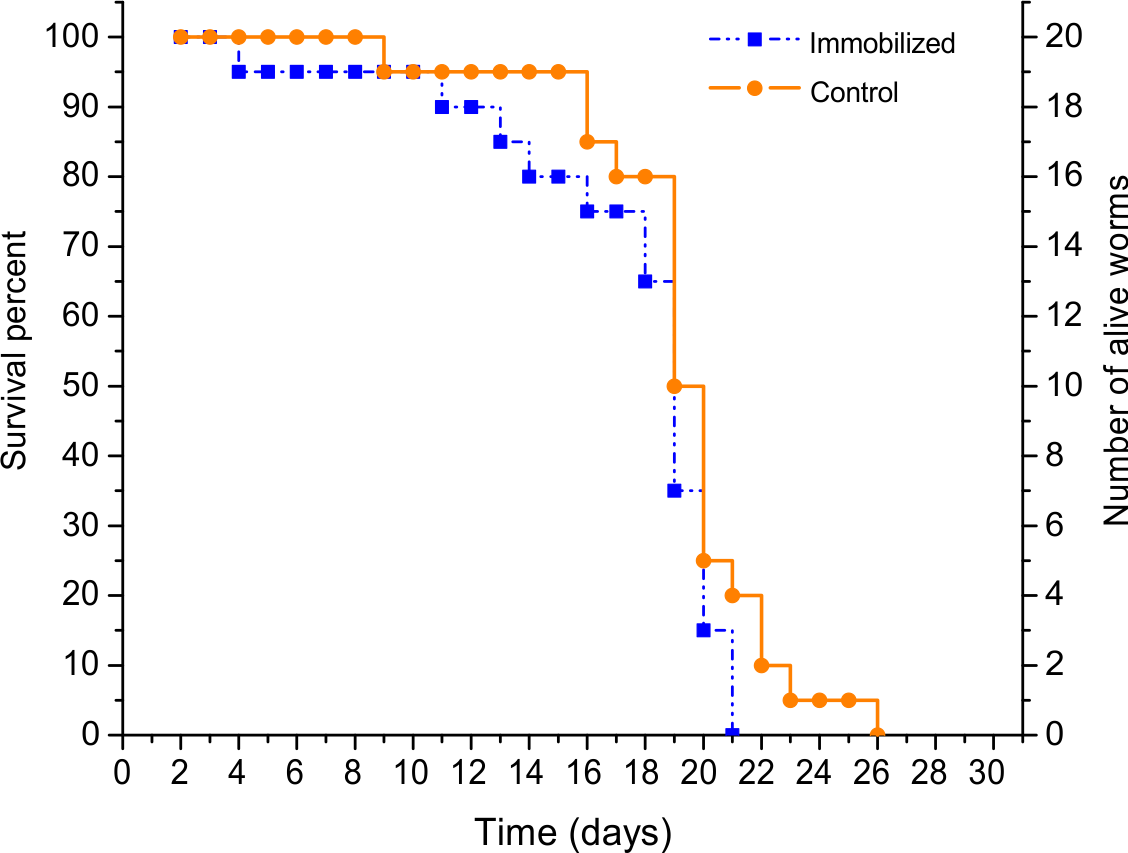

Supplement: Figure S5 — Lifespan analysis. The viability of worms immobilized automatically on the chip with an applied trapping pressure of 155 kPa for 30 seconds (blue) as compared to the control group (orange). (Log-Rank test, p = 0.14). (TIF) [file pone.0113917.s005.tif]

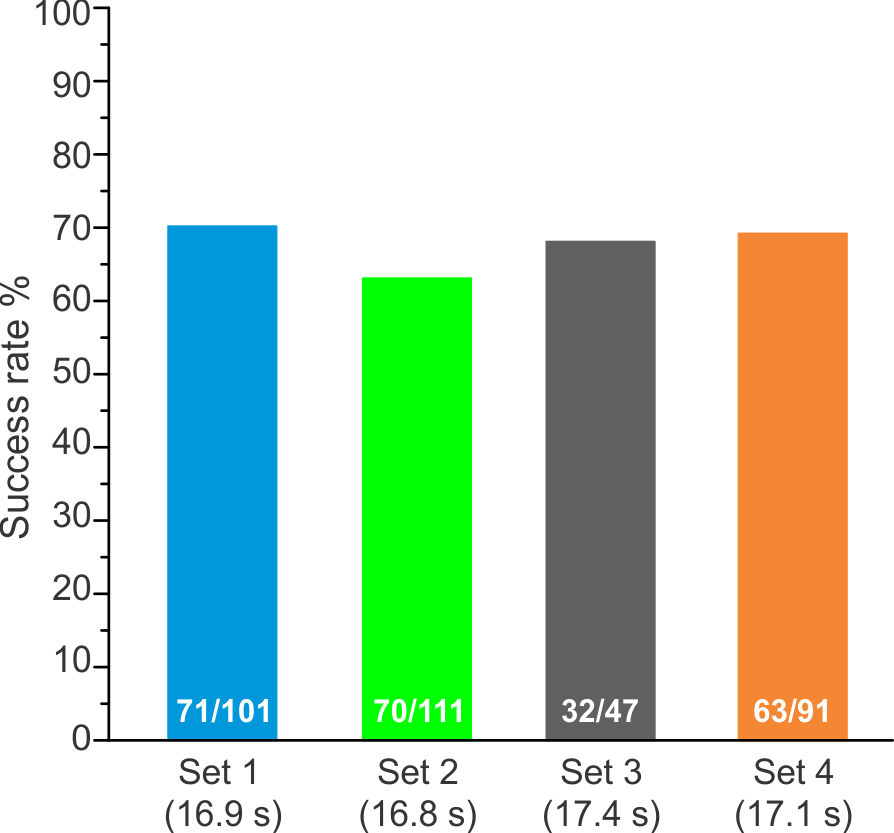

Supplement: Figure S6 — Statistical analysis of the automation steps. Four separate sets of automation experiments were pursued. Axons were severed successfully in 67.6±3.2% of the cases (n = 350). Actual number of processed worms and successfully severed worms are given inside each bar. Average full cycle process time for each set of experiments is given below the each bar in parenthesis. (TIF) [file pone.0113917.s006.tif]
