# Supplementary material for: A Fully Automated Microfluidic Femtosecond Laser Axotomy Platform for Nerve Regeneration Studies in C. elegans
Source: PLoS One. 2014 Dec 3;9(12):e113917. doi: 10.1371/journal.pone.0113917 (PMC4254741; doi:10.1371/journal.pone.0113917)
Supplement: File S1 — This supplementary document describes the automation software and the necessary hardware used for performing automated laser axotomy experiments described in the manuscript and provides the installation instructions of the software. (PDF) [file pone.0113917.s007.pdf]

Supplementary Software Document
for
A Fully Automated Microfluidic Femtosecond Laser Axotomy Platform for Nerve Regeneration Studies in *C. elegans*

Sertan Kutal Gokce[‡], Samuel X. Guo[†], Navid Ghorashian[ǂ], W. Neil Everett[†], Travis Jarrell[†], Aubri Kottek[†], Alan C. Bovik[‡], and Adela Ben-Yakar[†,ǂ,‡] *

[‡]Electrical and Computer Engineering, University of Texas, Austin, TX 78712
[†]Mechanical Engineering, University of Texas, Austin, TX 78705
[ǂ] Biomedical Engineering, University of Texas, Austin, TX 78705

Software Instructions and List of Hardware Requirements:

This manual describes the software used for controlling the microfluidic chip that was designed to perform automated laser axotomy experiments described in the original manuscript. This software is developed by Sertan Kutal Gokce, Sam Xun Guo, William Neil Everett, and Adela Ben-Yakar. The items that we include here are hardware list and screenshots of the LabVIEW front panels with a brief description of their operation. All software files can be found in compressed format at the following link:

https://research.engr.utexas.edu/benyakar/files/Automated_Axotomy_2014_Ver_4.rar

To install the software, the user has to extract the files in one folder and run **Axotomy.vi**. In the future, we may have different updates. To stay tuned, we welcome the readers to visit Ben-Yakar Group Website at:

https://research.engr.utexas.edu/benyakar/

The software is written in LabVIEW 2012. To properly run the software, the user must have LabVIEW 2012, LabVIEW Vision Acquisition Software, LabVIEW Vision Development Module, LabVIEW MathScript RT Module, NI Device Drivers, NI ELVISmx, µmanager (https://www.micro-manager.org/), and Matlab V2012a. This software is written specifically for running the microfluidic system presented in the paper, and uses efficient image processing algorithms for automated axotomy that is described in detail in the manuscript. This custom-software is developed to run specifically with the following hardware that was available in our lab:

1. Hardware list

Imaging and Ablation

- Camera: CoolSnap ES (Photometrics)
- Low-magnification objective: 5× air objective, Plan-Apochromat, NA=0.16 (Zeiss)
- High-magnification objective: 63× oil-immersion, Plan-Apochromat, NA=1.40 (Zeiss)
- Optical beam mechanical shutter : SH05 (Thorlabs)

* To whom correspondence should be addressed at: ben-yakar@mail.utexas.edu

- Optical beam mechanical shutter controller: SC10 (Thorlabs)

Fluidic control

- Three-way solenoid valves: LHDA0521111H (Lee Company)
- Valve control: Valvelink 8.2 (AutoMate Scientific)
- Digital solenoid valve control unit: NI-DAQ Card USB 6501 (National Instruments)

Position control

- Long-range motorized stages (3 axes): LTA-HS (Newport)
- Motorized stage controller (3 channels): 3 axes motion controller/driver, ESP300 (Newport)
- Piezo translational stages (3-axes): MAX 302 (Thorlabs)
- Open-loop piezo controller (3-channels): MDT693A, 3-Channel, open-loop piezo controller (Thorlabs)

Computer

- CPU: Core™ i5-2500K CPU @ 3.30 GHz (Intel)
- Graphics card: NVIDIA GeForce GT 520 (NVIDIA)
- Operating system: Windows XP Professional Version 2002 Service Pack 3 (Microsoft)
- Frame grabber: PCI LVDS Interface Card (Photometrics)

For different camera specs, translational stages, communication settings, and/or microscope objectives' specs, modifications to the program will be necessary to control and accommodate for the different hardware specs.

In the following sections we will describe the details of the LabVIEW software and the associated panels.

2. Axotomy.vi

This is the main LabVIEW VI (Virtual Instrument) that controls all of the hardware listed above, performing automated axotomies, and defining all the axotomy parameters used for automation. The front panel consists of eight main tabs, as shown in Figure 1:

1. START
2. CCD Camera
3. Motorized Stage
4. Shutter
5. Piezo Stage
6. Valves
7. ROI Locations
8. Automated Process

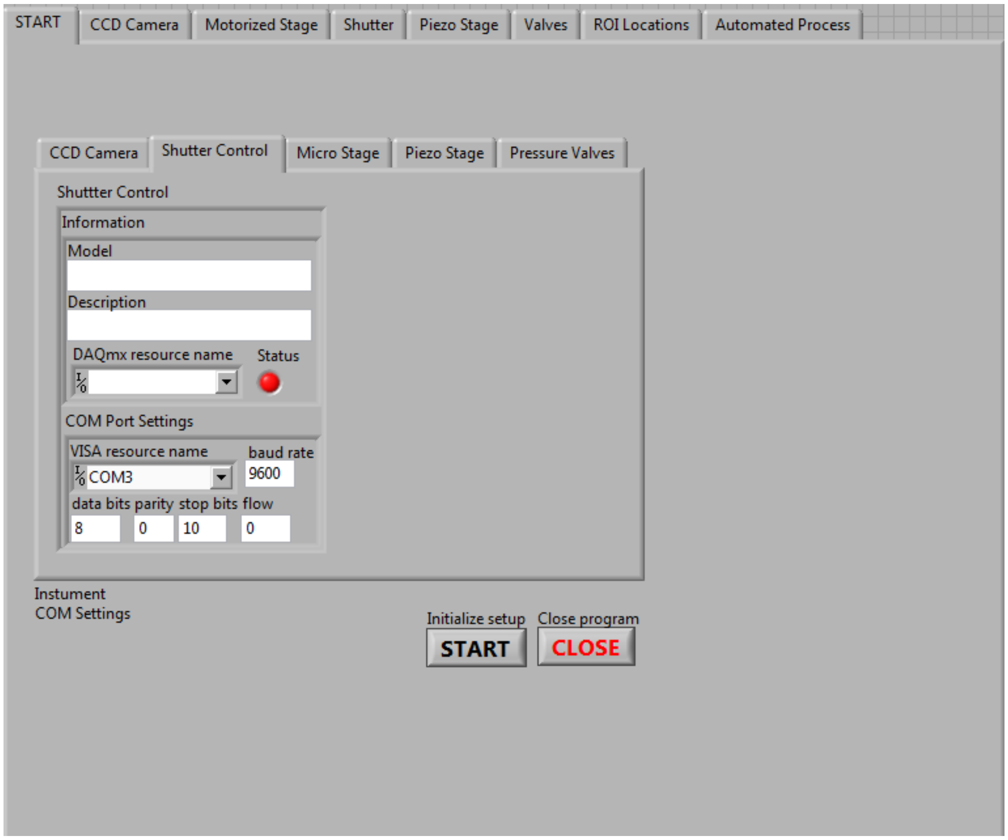

Figure 1: The front panel of Axotomy.vi with "START" tab chosen.

Each tab panel is used for controlling different hardware components and automation parameters. The brief details of each tab is given in the following sections.

2.1. START tab (Figure 1): This tab is used to initiate communication with all the hardware components by simply clicking on the "START" button. After clicking the start button, the computer communicates with each associated hardware to acquire the status and communication settings of each hardware. Each sub-tab then shows the status (false/correct) of each associated hardware whether there is a communication problem between hardware and program. If the user is using a different hardware, the communication settings may have to be changed in the block diagram of the program.

2.2. CCD Camera tab (Figure 2): This tab is where we indicate the camera exposure times for each magnification and the image file saving options for the automated process. When the program is used manually, the user can change the exposure time to a desired value by using the numerical control on the left side of the panel in the "Exposure time" section. The user may choose the file format of the saved images by changing the parameters in the middle section called "Save options for CCD Camera Images and Log File". For the automation process, the user has to define exposure times for both FOVs in the right section named "Automated Process Exposure Times".

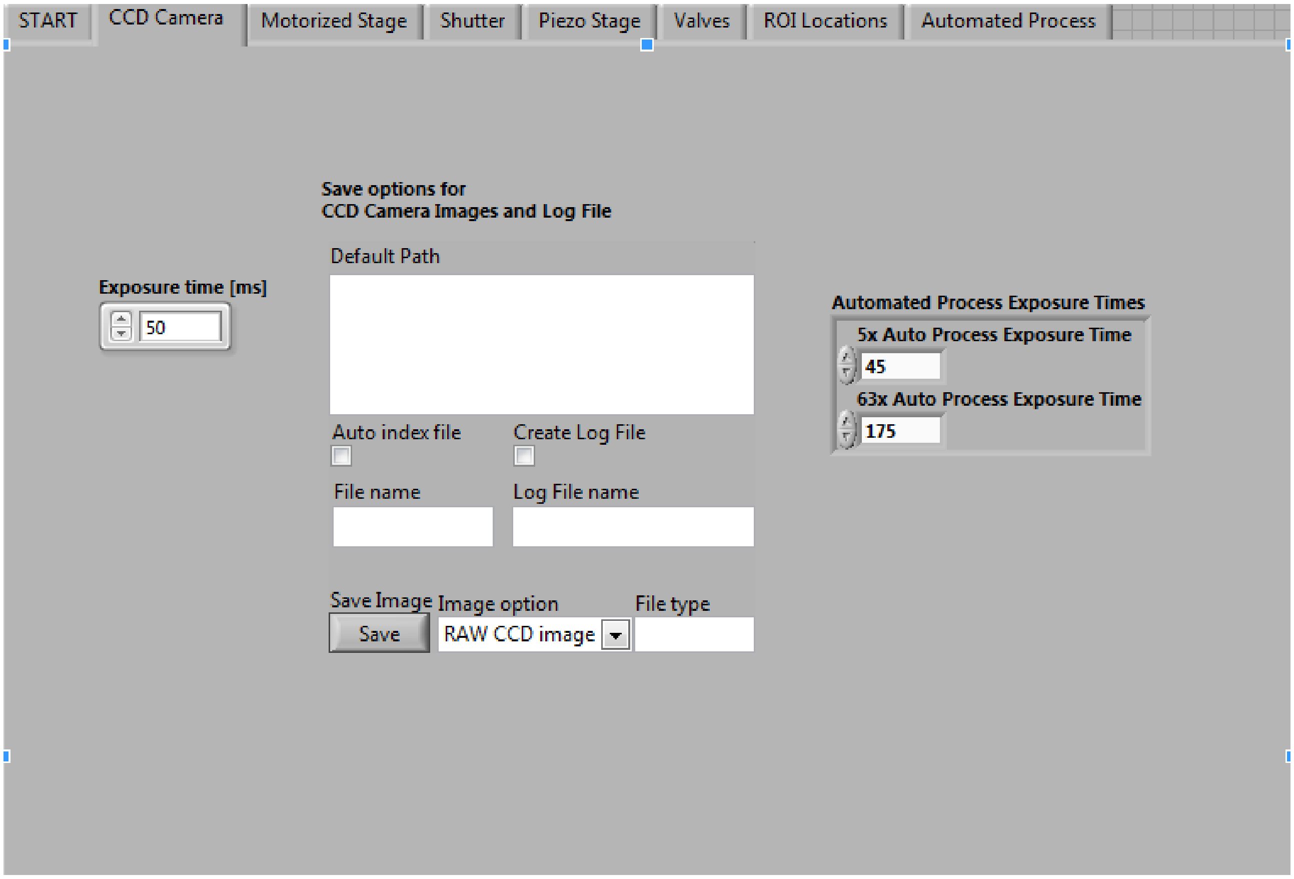

Figure 2: The front panel of Axotomy.vi with "CCD Camera" tab chosen.

2.3. Motorized Stage tab (Figure 3): This tab incorporates control parameters of the long-range translational stage that are entered manually before starting the automated process as well as for manual manipulation. To run the axotomies automatically, the operator needs to define only one parameter manually: the initial position of the stage as called "Home Position". To define this parameter, the user needs to move the specimen to a desired location close to the immobilization area and save that location as the home position by choosing "Home Position" item in the section called "Saved Positions Micro". This home position serves as the initial rest position of the stage before each cycle of the automation process. The operator can also store five more preset locations (in the section "Saved Position Micro") during manual actuation for moving the stage to additional preset locations. During manual operation, the operator can move the specimen along three axes (x, y, z) by inserting the desired parameters for each axis such as acceleration, speed, and backlash compensation in the left section called "Micro Stage Data". Manual movement of the stage can also be executed by using the graphical XY plane and Z control cursor at the bottom of the panel.

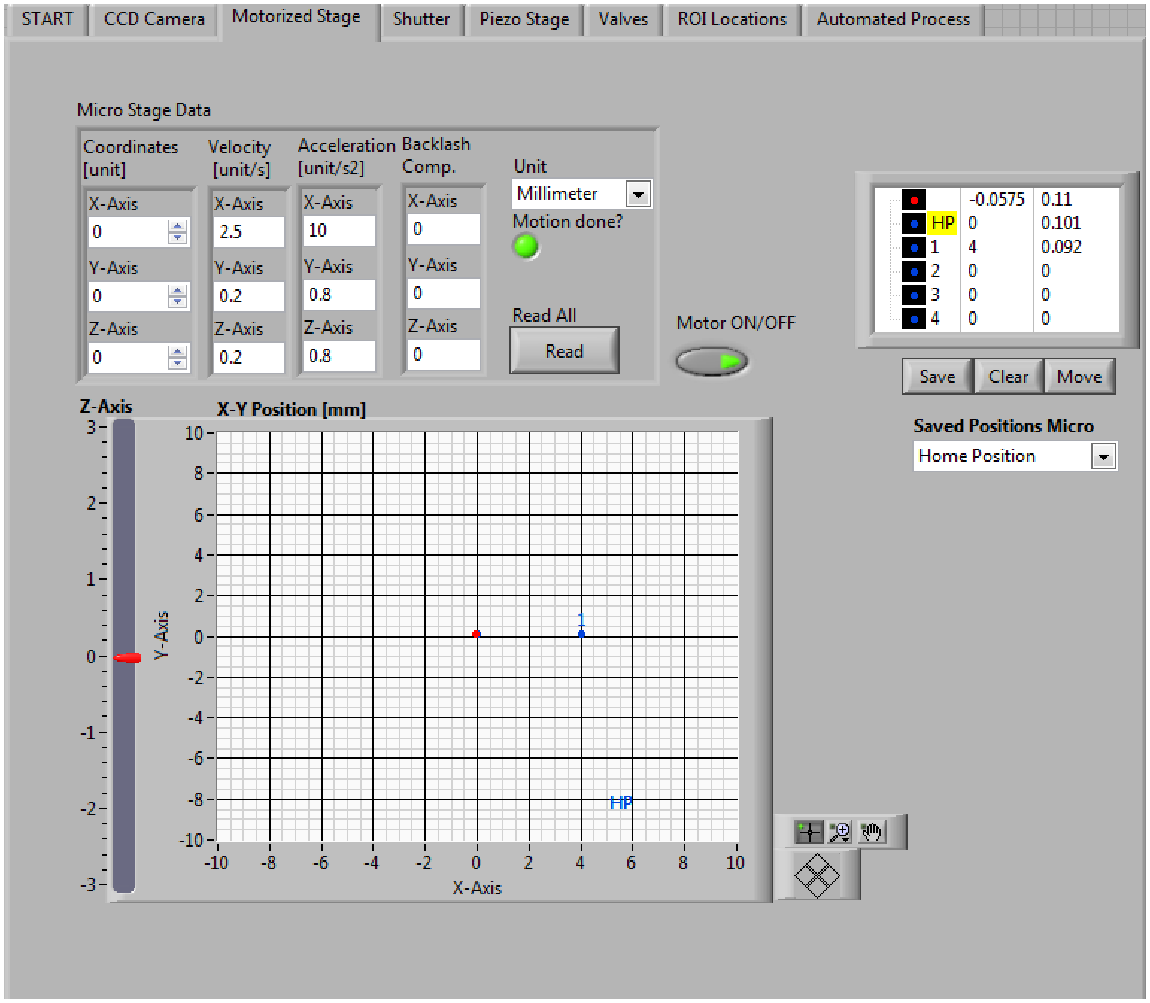

Figure 3: The front panel of Axotomy.vi with "Motorized Stage" tab chosen.

2.4. Shutter tab (Figure 4): This tab defines the settings of the mechanical shutter for the laser surgery beam. In the “Shutter Settings” section, the user may choose between three different shutter modes (Single, Auto, and Manual) and adjust the shutter opening/closing times. The description of each shutter toggle modes are described below.

- **Single:** In the “Single” event mode the shutter will open and close with a click on the “Send the beam” Boolean control. In this mode the shutter will remain open for the duration defined in the “Open time [ms]” and then close automatically.
- **Auto:** In the “Auto” mode the shutter will continually open and close after clicking the “Send the beam” Boolean control based on the parameters defined in “Open/Close time” windows.
- **Manual:** In manual mode the shutter will be open with a click of the “Send the beam” Boolean control and will stay open till the Boolean control is clicked again.

For the automated axotomies, the operator needs to choose the “Single” mode and define opening time of the shutter in the “Open time [ms]” section. We usually use an opening time of 300 ms, letting 300 laser pulses pass through the mechanical shutter as they are generated at 1 kHz repetition rate. For manual axotomies, the user needs to press the “Send the beam” button to set up desired shutter modes such as continuous or single-shot mode. “Send the beam” button is only used during manual manipulations.

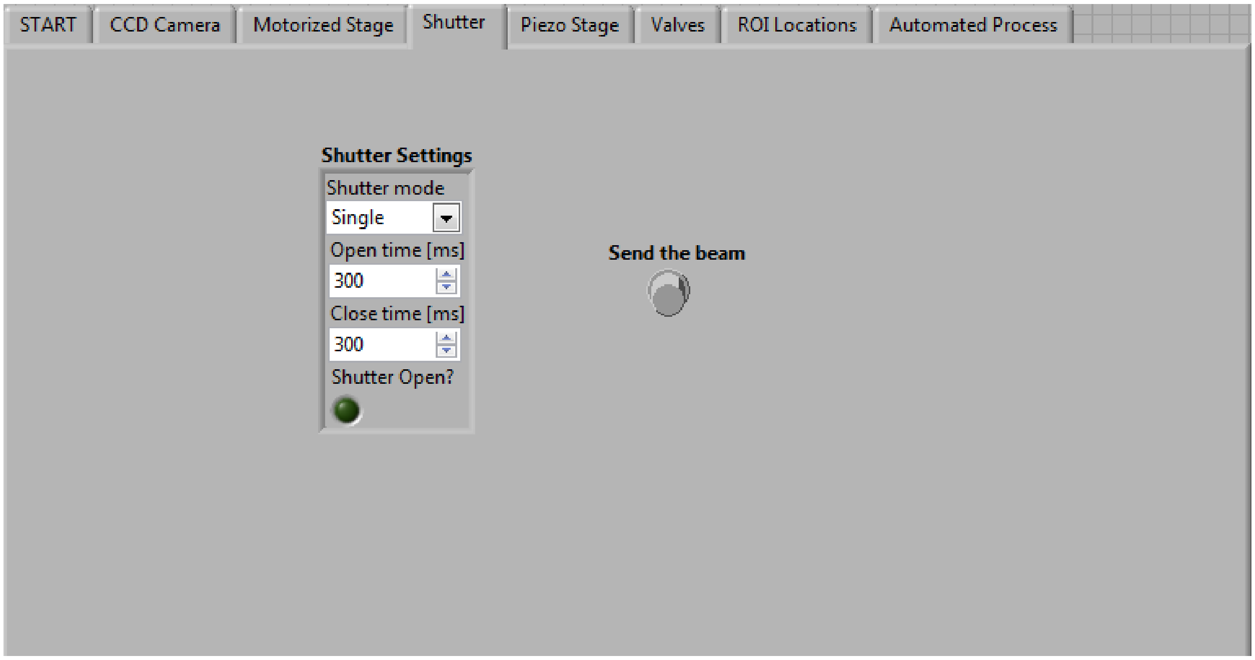

Figure 4: The front panel of Axotomy.vi with “Shutter” tab chosen.

2.5. Piezo Stage tab (Figure 5): This tab allows the user to control manually the input voltage range for the piezo driver in the sections of “Min Voltage Piezo” and “Max Voltage Piezo”. The “Coordinates Piezo Stage [um]” is used to move the piezo stage manually and “Get the position” section is used to read current position of the stage.

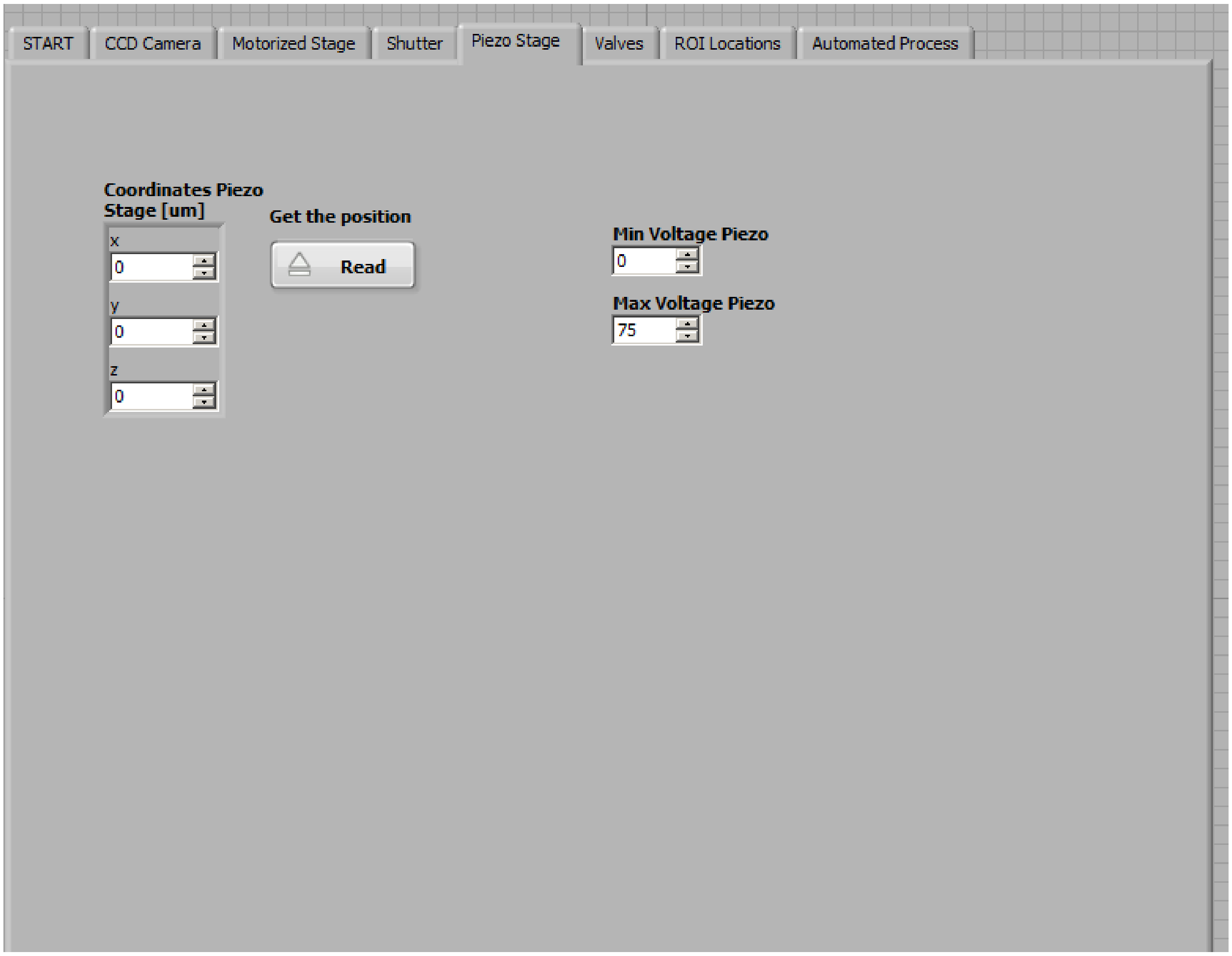

Figure 5: The front panel of Axotomy.vi with “Piezo Stage” tab chosen.

2.6. Valves tab (Figures 6-12): This tab accesses the state of individual valves and the timing and flow scheme of in each of the sequences in the following sub-tabs:

- Main Valve Control (Figure 6)
- Pre-Loading Sequence (Figure 7)
- Injection Sequence (Figure 8)
- Immobilization Sequence (Figure 9)
- Ejection Sequence (Figure 10)
- Flushing Sequence (Figure 11)
- Cleaning Sequence (Figure 12)

The "Main Valve Control" sub-tab is used to change individual state of each solenoid valve control and run the flow schemes defined in the adjacent sub-tabs. The "Main Valve Control" tab also allows user to save and load the user-defined flow schemes in TDMS file format. The valve sequence ("Automated Axotomy Flow.tdms") used for this study is provided with the compressed downloadable program folder.

There are six flow schemes defined in the sub-tabs (Pre-Loading Sequence, Injection Sequence, Immobilization Sequence, Ejection Sequence, Flushing Sequence and Cleaning Sequence). The first five of these sub-tabs are used in different steps of the automation sequence. The "Pre-Loading Sequence" is used to isolate one single worm from the loading area (see Figure 7), by opening the Valve 6 for 850 ms automatically for each cycle of automation. The "Injection Sequence" is used to inject the isolated worm during the pre-loading sequence for immobilization. The "Immobilization Sequence" is used to pressurize the trapping membrane for taking background image during the automation Step 1 (see Figure 9). The "Ejection Sequence" and "Flushing Sequence" are used to transport the processed animals to two different outlets based on whether automation cycle is successful or not (see Figures 10 & 11) by opening valves V3, V2, and V4 or V5, and turning on the inlets of Pillar and Flush. The sixth and last tab includes the "Cleaning Sequence"; which is used to clean the chip by opening all the valves and turning on all the flow inlets whenever desired by clicking the corresponding Boolean controls on the "Main Valve Control" sub-tab. The order of valve steps in the schemes are saved as an array of Boolean clusters. The order of these steps is identified by the elements of the array starting n=0 (upper left control in each scheme). For example, the first step of the injection sequence consists of turning on the side injection and opening valves V1 and V3 for 1000 ms as zeroth element (see Figure 8a). In each of the "Sequence" sub-tabs, the user can input timings for each valve state combination to achieve desired functionality.

For individual control of each solenoid valve, there are two extra controllers defined in case the operator would like to incorporate additive valve controls into the system (see dashed circle in Figure 6).These additional valves can be controlled after easily defining two digital outputs via NI

DAQ MX . Control of individual valves can also be carried out using Boolean image cluster shown as “Valves Image Cluster” at the bottom of “Main Valve Control” sub-tab (Figure 6). The Boolean controls are placed on the picture of the axotomy chip in accordance with the actual relative locations of the valves and inlets on the chip. The closed control valves are defined by a “red X” and the open valves are left blank or defined by a “blue arrow” in the case of I/O (input or output).

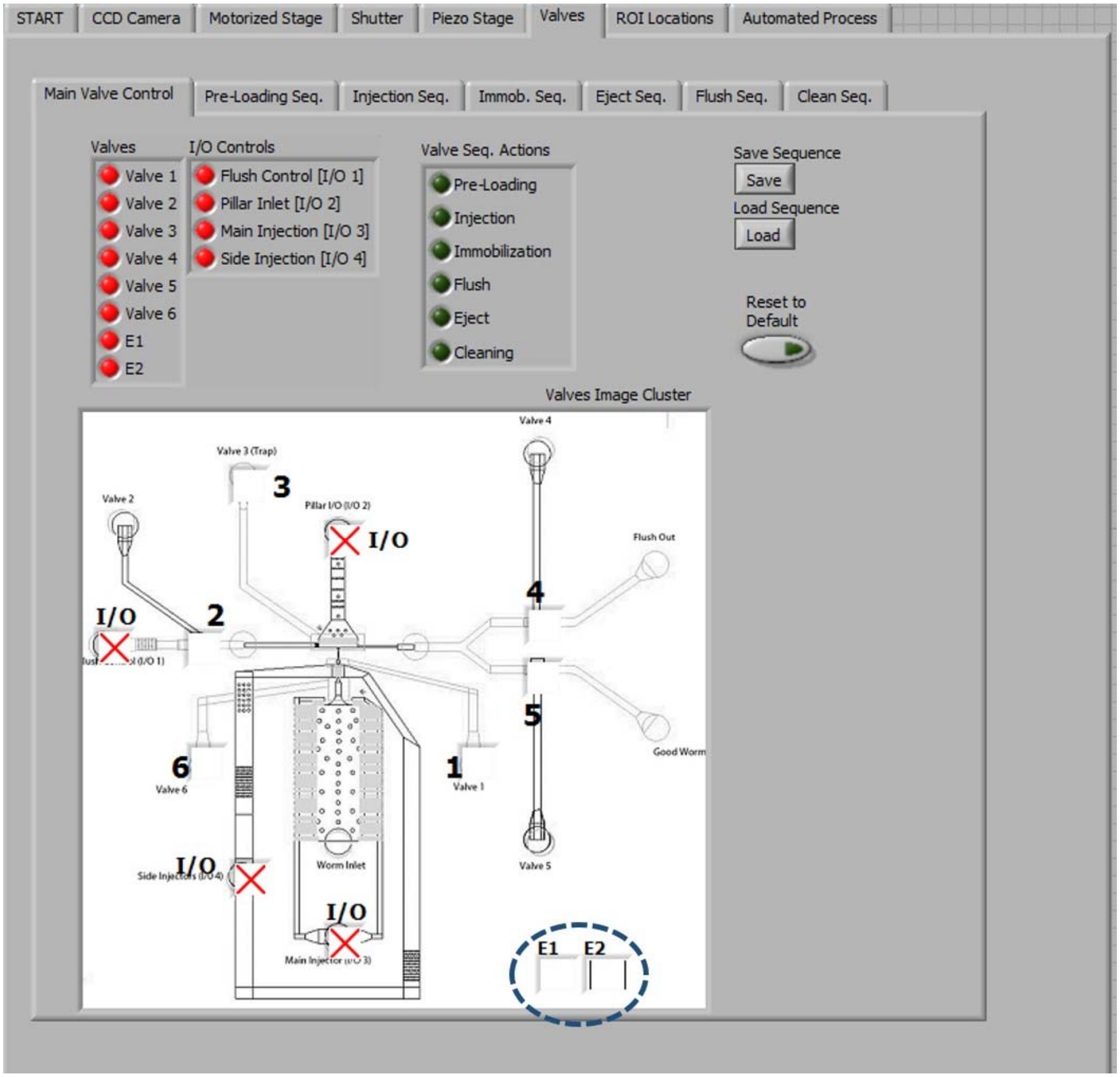

Figure 6: The front panel of Axotomy.vi with “Valves” tab and “Main Valve Control” sub-tab chosen.

a)

b)

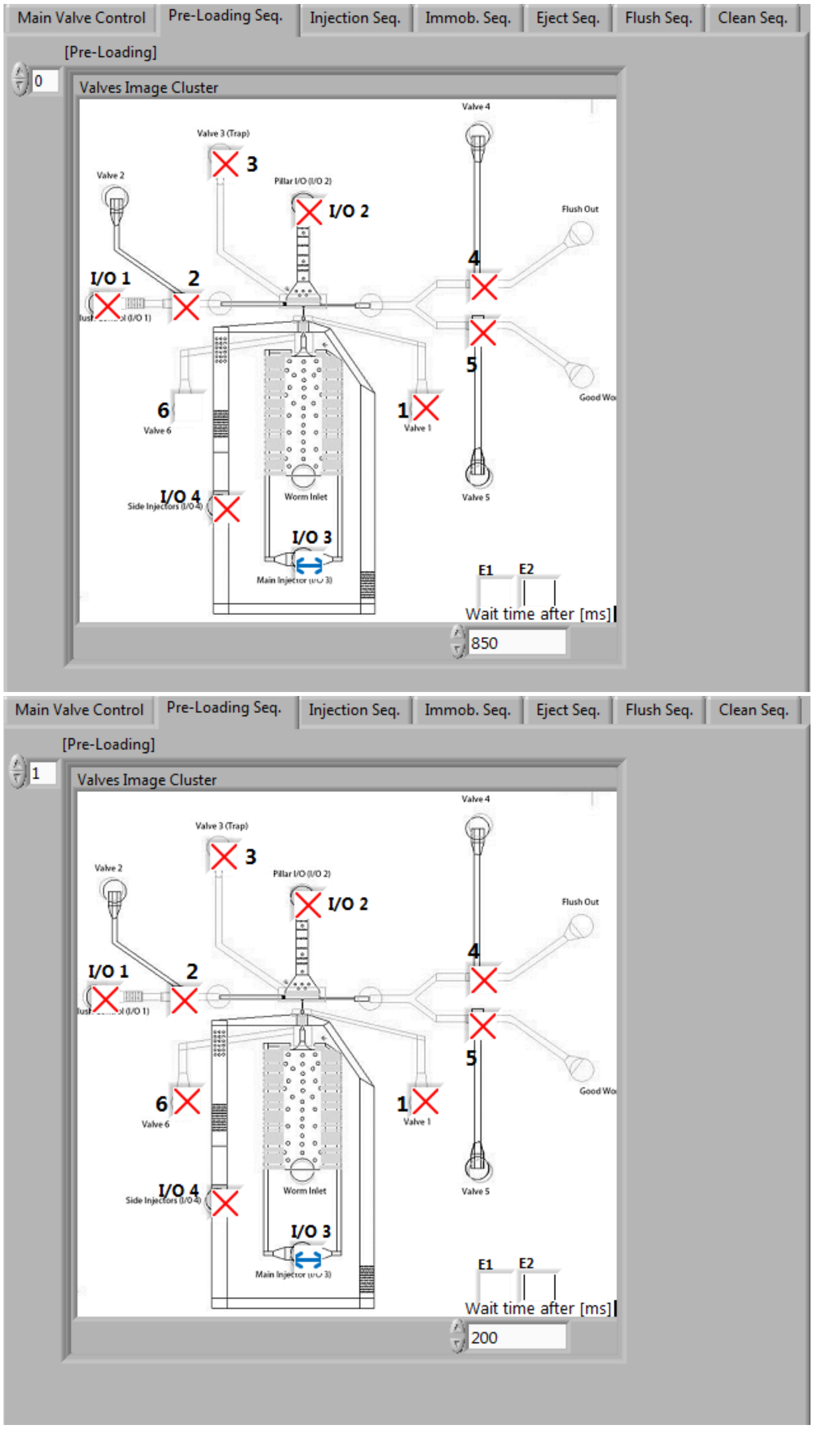

Figure 7: Two elements of Pre-Loading Sequence: a) Element 0: To stage a single worm from the loading chamber into the staging area by opening Valve 6 for 850 ms. b) Element 1: Default valve state.

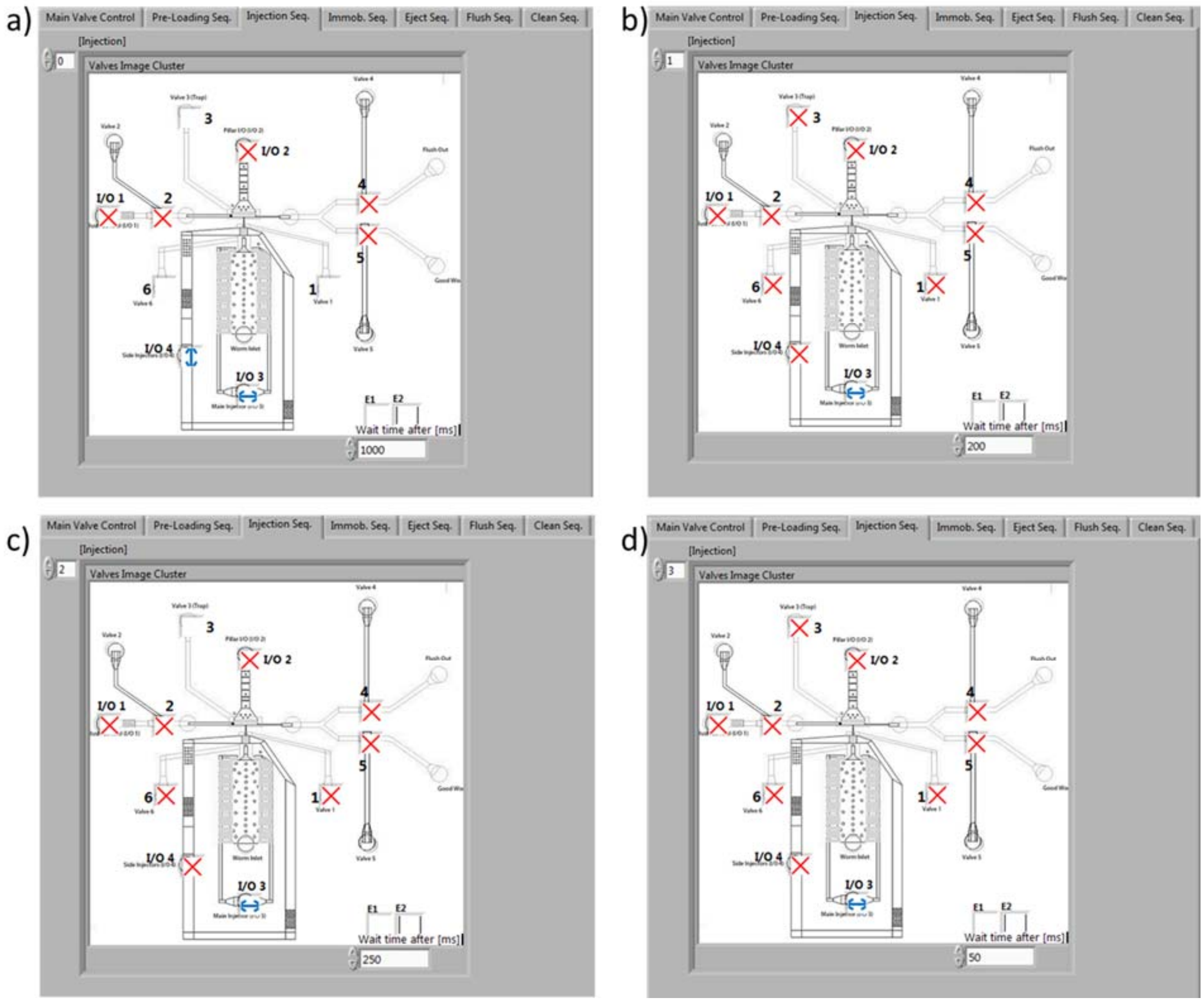

Figure 8: Four elements of the Injection Sequence: a) Element 0: Injecting the worm into the trapping area and pushing the extra worms back into loading chamber by opening Valve 1 and Valve 6 and reversing the flow in the staging side channels for 1000 ms. b) Element 1: Valve 3 is opened and c) Element 2: Valve 3 is closed in a pumping manner as described in the main manuscript before going back to default state in d) (Element 3).

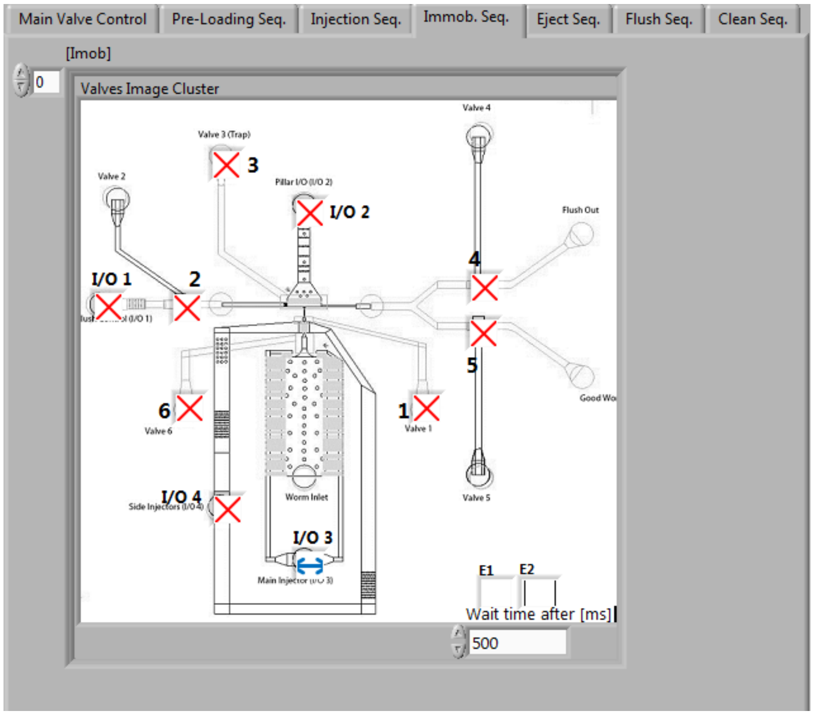

Figure 9: Immobilization Sequence: This sequence is same with the default state where Valve 3 remains closed immobilizing the worm – so there is only one element in this sub-tab. The software waits for 500 ms in the automation sequence during image subtraction process (Step 1).

a)

b)

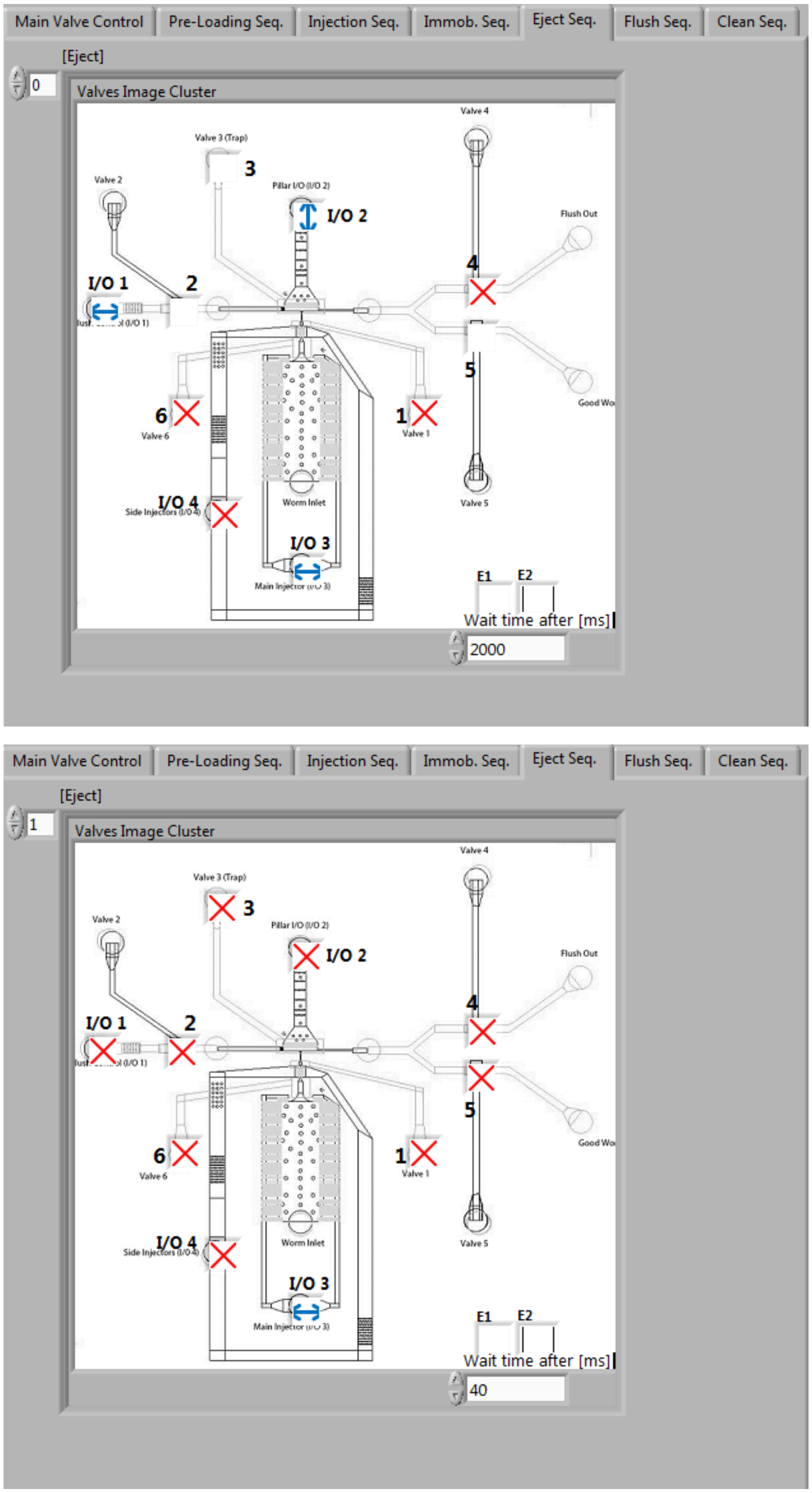

Figure 10: Two Elements of Ejection Sequence: a) Element 0: Transporting the successfully axotomized worm into the recovery area through the ejection channel by turning on Flush I/O and Pillar I/O and turning off Valve 2, Valve 3, and Valve 5 for 2000 ms. b) Element 1: Returning back to default state.

a)

b)

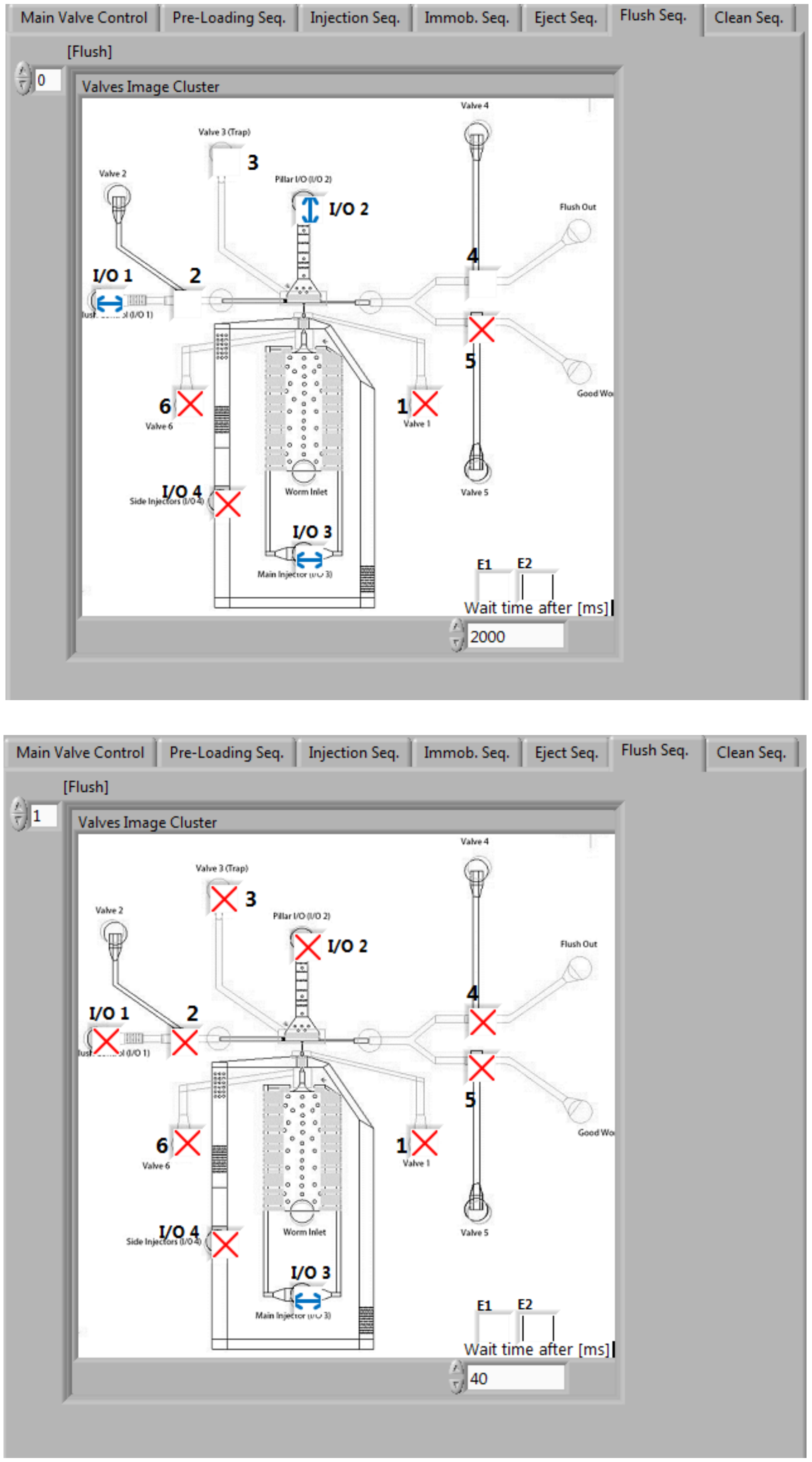

Figure 11: Two Elements of Flush Sequence: a) Element 0: Transporting the unsuccessfully processed worm into the waste outlet through the ejection channel by turning on Flush I/O and Pillar I/O and turning off Valve 2, Valve 3, and Valve 4 for 2000 ms. b) Element 1: Returning back to default state.

a)

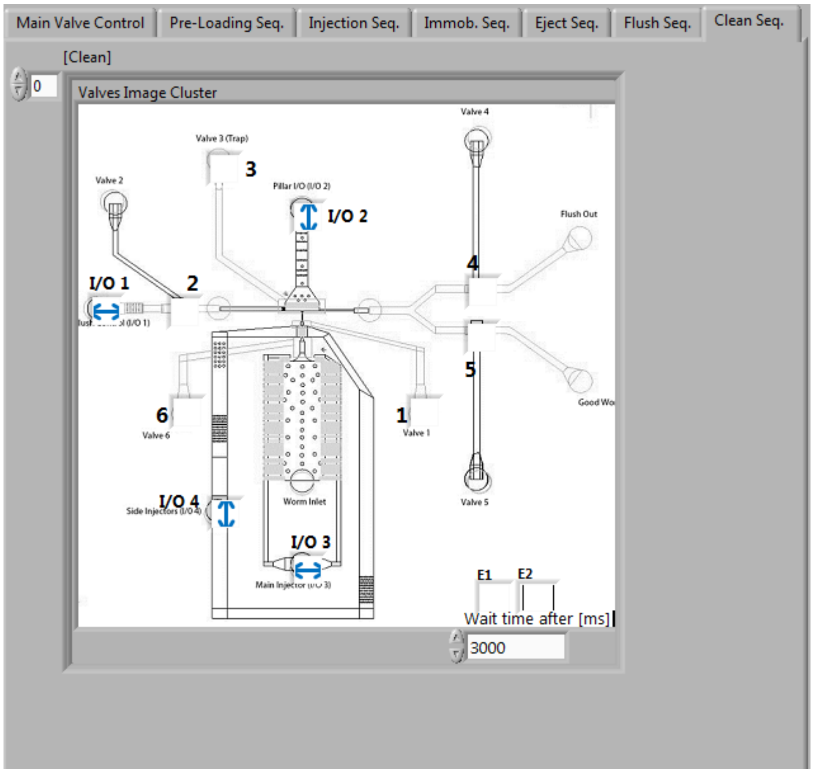

b)

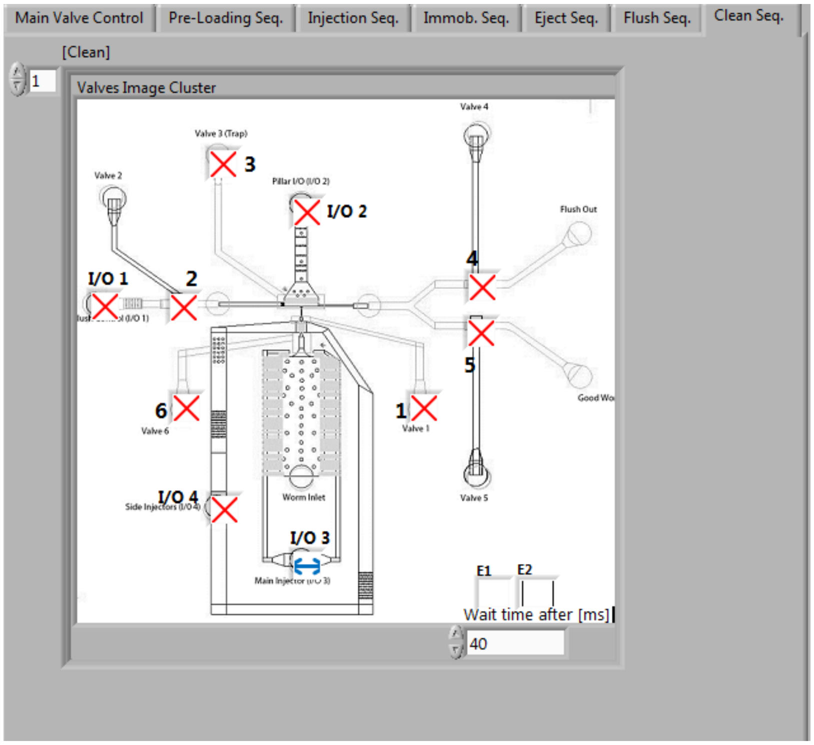

Figure 12: Elements of Cleaning Sequence: Element 0: a) Turning on each flow inlets and de-pressurizing all control valves for 3000 ms. b) Element 1: Returning back to default state.

2.7. ROI Locations (Figure 13): This tab is used for inputting information about the region-of-interests (ROI), which involves the femtosecond laser spot (the details of how to find the spot are described in the manuscript) and immobilization chamber (pre-defined ROI for Step 1 of the automation process). The ROI information should be defined before starting the automation process.

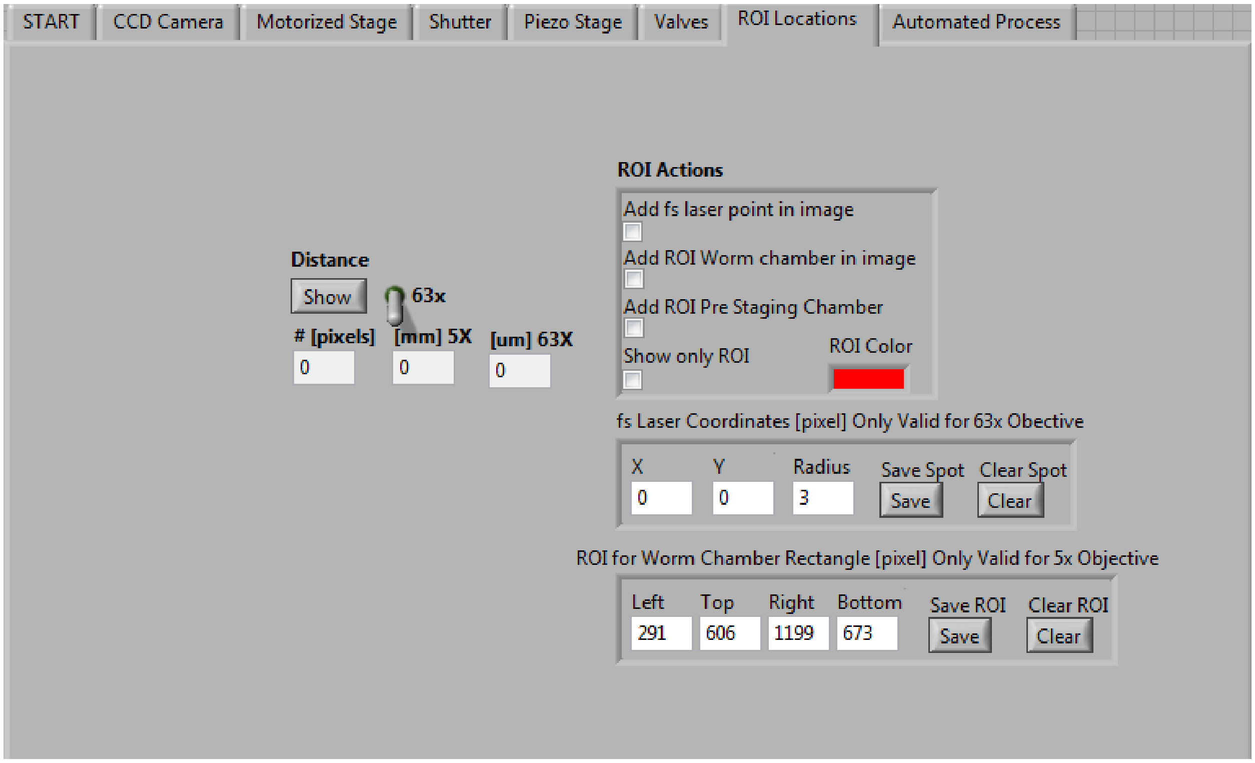

Figure 13: The front panel of Axotomy.vi with "ROI Locations" tab chosen.

2.8. Automated Process tab (Figure 14): This tab allows the user to initiate the automated axotomy process by clicking the "Start" button of the Boolean control "**Start the automated process"**. This tab also allows the user to choose the number of worms to be processed, types of images to be saved throughout the automation process, and the offset between the focal planes of two objectives using this tab. Each of the image processing algorithms used for the automation is described explicitly in the paper. The automation algorithm flow, also described in the main manuscript explicitly (see also Figure S3), is executed automatically in the background for the desired number of worms to be processed. The success/failure of each step is saved automatically for each step.

Before the automation process starts, the user has to define the following parameters:

- File saving location.
- Home position for motorized stage.
- Desired shutter settings.
- Valve flow scheme and corresponding timing.
- ROI locations for femtosecond laser spot and immobilization chamber.
- Selection of images to be saved at different steps of the automation process.
- Number of worms to be processed and z-offset between the focal planes of the low- and high-magnification objectives.

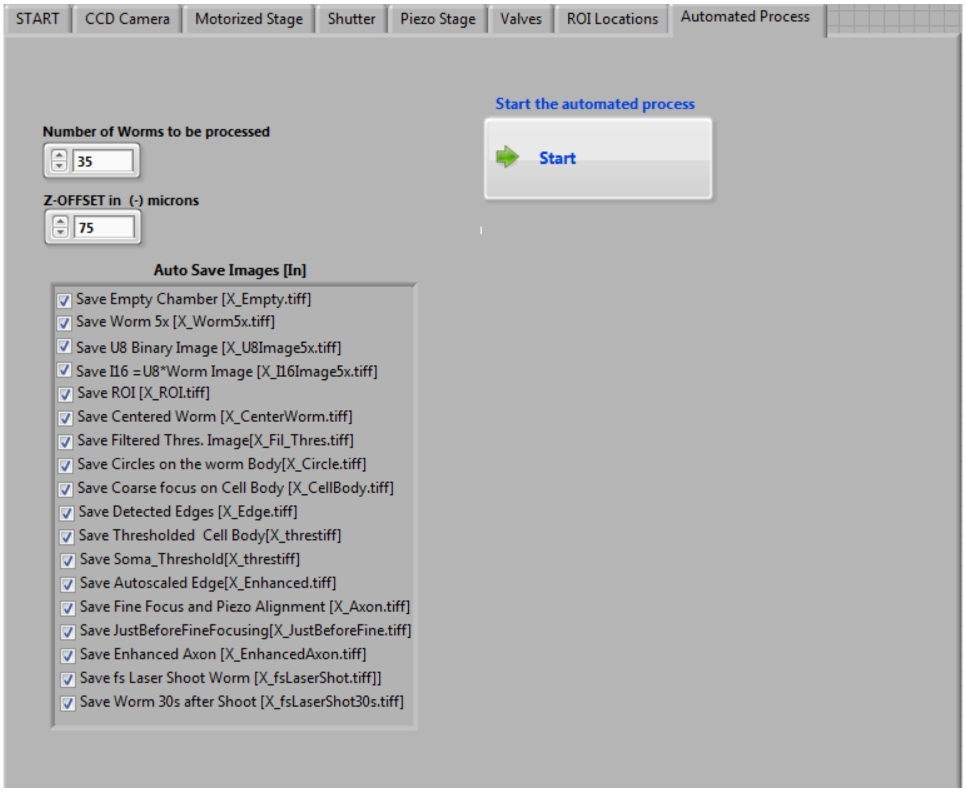

Figure 14: The front panel of Axotomy.vi with "Automated Process" tab chosen.
